# ﻿A critical review on cytogenetics of Cucurbitaceae with updates on Indian taxa

**DOI:** 10.3897/compcytogen.v16.i2.79033

**Published:** 2022-04-26

**Authors:** Biplab Kumar Bhowmick, Sumita Jha

**Affiliations:** 1 Department of Botany, Scottish Church College, 1&3, Urquhart Square, Kolkata-700006, West Bengal, India Scottish Church College Kolkata India; 2 Plant Cytogenetics and Biotechnology Laboratory, Department of Botany, University of Calcutta, 35, Ballygunge Circular Road, Kolkata 700019, West Bengal, India University of Calcutta Kolkata India

**Keywords:** chromosome, genome size, karyotype, NORs, ploidy

## Abstract

The cytogenetic relationships in the species of Cucurbitaceae are becoming immensely important to answer questions pertaining to genome evolution. Here, a simplified and updated data resource on cytogenetics of Cucurbitaceae is presented on the basis of foundational parameters (basic, zygotic and gametic chromosome numbers, ploidy, genome size, karyotype) and molecular cytogenetics. We have revised and collated our own findings on seven agriculturally important Indian cucurbit species in a comparative account with the globally published reports. Chromosome count (of around 19% species) shows nearly three-fold differences while genome size (of nearly 5% species) shows 5.84-fold differences across the species. There is no significant correlation between chromosome numbers and nuclear genome sizes. The possible trend of evolution is discussed here based on molecular cytogenetics data, especially the types and distribution of nucleolus organizer regions (NORs). The review supersedes the scopes of general chromosome databases and invites scopes for continuous updates. The offline resource serves as an exclusive toolkit for research and breeding communities across the globe and also opens scope for future establishment of web-database on Cucurbitaceae cytogenetics.

## ﻿Introduction

The family Cucurbitaceae contains an extensive range of diversity consisting of about 1000 species spread over 96 genera (Renner and Schaefer 2017). The diversity of plant families is associated with variation in genome sizes and chromosome numbers as a result of enormous adaptive radiation (Soltis et al. 2004; Lysák and Schubert 2013). The viewpoint of evolution has been changed with the understanding of whole genome duplication (WGD) (Soltis et al. 2014) followed by core-eudicot hexaploidy (Wang et al. 2018). A cytogenetic database is essential to gain insights into evolution by supplementing phylogeny trees with chromosome number information (Mota et al. 2016) to upgrade knowledge on plant systematics (Soltis et al. 2014; Viruel et al. 2021). Cucurbitaceae, being the fourth most important and one of the earliest consumed vegetables yielding family, has coped with extreme climates, extensive human intervention and a huge domestication syndrome (Chomicki et al. 2020). Considerable advances have been made in molecular phylogeny (Renner and Schaefer 2016; Bellot et al. 2020; Chomicki et al. 2020; Guo et al. 2020) and genomics (CuGenDB, http://cucurbitgenomics.org) (Zheng et al. 2019). We had previously discussed about the gaps in cytogenetic studies (Bhowmick and Jha 2015b) which has been surmounted with the advent of molecular cytogenetics.

Currently, we have collated the cytogenetic reports of Cucurbitaceae globally and integrated our own findings for a collective interpretation. The review attempts to address i) the trend of chromosome evolution in specific tribes and species based on available information, ii) correlation between chromosome numbers and ploidy or genome size in the studied taxa and iii) the requirement of an exclusive cytogenetic catalogue for genome researchers, taxonomists and breeders working on Cucurbitaceae.

## ﻿Methodological approaches

### ﻿Data compilation

The data have been collated as per Schaefer and Renner (2011) after consultation of books, Chromosome atlases, research articles and public resources like Chromosome Counts Database (CCDB; http://ccdb.tau.ac.il/) (Rice et al. 2015), The Index to Plant Chromosome Numbers (IPCN, http://legacy.tropicos.org/Project/IPCN) (Goldblatt and Lowry 2011) and The Plant DNA C-values database (Pellicer and Leitch 2020) (https://cvalues.science.kew.org/).

### ﻿Chromosome analysis in the Cucurbit species ocurring in India

Presently an enzymatic maceration and air drying (EMA) method followed by flurochrome banding has been employed as per our previous protocols (Bhowmick et al. 2012, 2016; Bhowmick and Jha 2015a, 2019, 2021) to represent fresh karyotypes of seven agriculturally important cucurbit species (Table 1) belonging to Benincaseae and Sicyoeae. Fresh and healthy roots were used from different sources (like germinating seeds, seedlings and underground root stocks). Roots were pretreated with 0.002 M hydroxyquinoline and fixed in 1:3 aceto-methanol solution. The standardization of EMA- fluorescence banding was conducted for the different species. In brief, fixed roots were digested in enzyme mixture [1% Cellulase (Onozuka RS), 0.75% Macerozyme (R-10), 0.15% Pectolyase (Y-23), 1 mM EDTA] for 40–45 min at 37 °C, macerated on slides, air-dried, stained with 2% Giemsa solution (Merck, Germany) and plates selected for karyotyping. After de-staining, slides were kept in McIlvaine buffer, stained with 0.1 µg mL^-1^ DAPI for 15–20 min in darkness. For CMA staining, slides were incubated in 0.1 mg mL^-1^ CMA for 15–25 min in darkness. For meiotic chromosomes, fixed anthers were digested in enzyme mixture for 5–8 min, macerated on slides and DAPI staining protocol was followed with minor modifications. All slides were mounted in non-fluorescent glycerol and chromosome plates were observed under a Zeiss Axioscop 2 fluorescence microscope (using UV and BV filter cassettes for DAPI and CMA stains, respectively). Images were captured using the attached ProgRes MFscan Jenoptik D07739 camera and ProgRes CapturePro 2.8.8 software.

**Table 1.** Chromosome numbers and nature of fluorescent bands in some cucurbit species occurring in India.

Tribes	Species (common name, status of cultivation/ wild)	Collection site, Latitude/ Longitude	Fruit image	2n	CMA bands		DAPI bands (Non-nucleolar)
					Nucleolar	Non-nucleolar	
**Sicyoeae**	*Luffaacutangula* Linnaeus, 1753 (ridged gourd, cultivated)	Bhubaneswar, Odisha, 20.2960°N, 85.8245°E	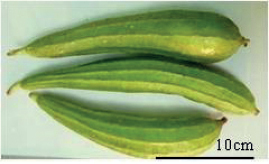	26	11^th^ , 12^th^, 13^th^	12^th^ (centromeric)	1^st^ to 13^th^ (distal)
	*Luffacylindricaaegyptiaca* Miller, 1768 (sponge gourd, cultivated)	Imphal, Manipur, 24.6637°N, 93.906°E	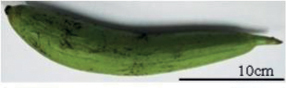	26	12^th^ , 13^th^	1^st^ , 2^nd^ (distal)	0
	*Luffaechinata* Roxburgh, 1814 (wild)	Pantnagar, Uttarakhand, 30.0667°N, 79.019°E	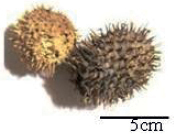	26	11^th^ , 12^th^ , 13^th^	0	1^st^ to 13^th^ (distal)
	*Trichosanthescucumerina* Linnaeus, 1753 (wild)	NBPGR, Thrissur, Kerala, 10.5276°N, 76.2144°E	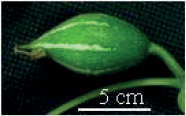	22	10^th^ , 11^th^	0	1^st^ to 11^th^ (distal)
	Trichosanthescucumerinassp.cucumerina Anguina (snake gourd, cultivated)	Bengaluru, 12.9716°N, 77.5946°E	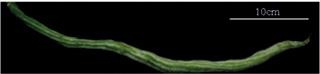	22	10^th^ , 11^th^	2^nd^ (distal)	0
	*Trichosanthesdioica* Roxburgh, 1832 (pointed gourd, cultivated)	Bhagalpur, Bihar, 25.2414°N, 86.9924°E	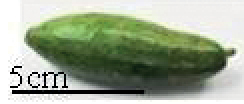	22 (female)	0	7^th^ , 8^th^ , 10^th^ (distal)	1^st^ to 11^th^
			22 (male)	0	0	1^st^ to 11^th^
**Benincaseae**	*Benincasahispida* Thunberg, 1784 (ash gourd, cultivated)	Imphal, Manipur 24.6637°N, 93.906°E	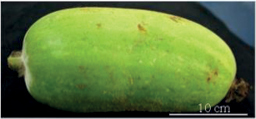	24	12^th^	9^th^ (distal)	0
	*Cocciniagrandis* Linnaeus, 1767 (ivy gourd, restricted cultivation)	Nagpur, Maharashtra, 21.1458°N, 79.0881°E	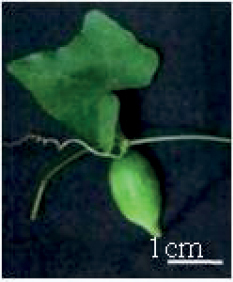	24 (female)	8^th^, 12^th^ *	1^st^ to 5^th^, 8^th^ to 12^th^ (centromeric)	0
			24 (male)	8^th^, 12^th^ *	1^st^ to 5^th^, 8^th^, 10^th^ to 12^th^ (centromeric)	0

### ﻿Statistical analyses

Statistical analysis involving foundational cytogenetic parameters have been demonstrated to imply significant knowledge on chromosomal evolution within a group (Winterfeld et al. 2020). Considering the lack of hypotheses, we have tested for correlation between the dependent variables (2C genome size, MCL and HCL) and predictor variables [chromosome number (2n) and ploidy level (pl)] and also calculated linear models for regression analysis using IBM SPSS (v23, free).

### ﻿The modern cytogenetic catalogue of cucurbitaceae

**Figure 1.**
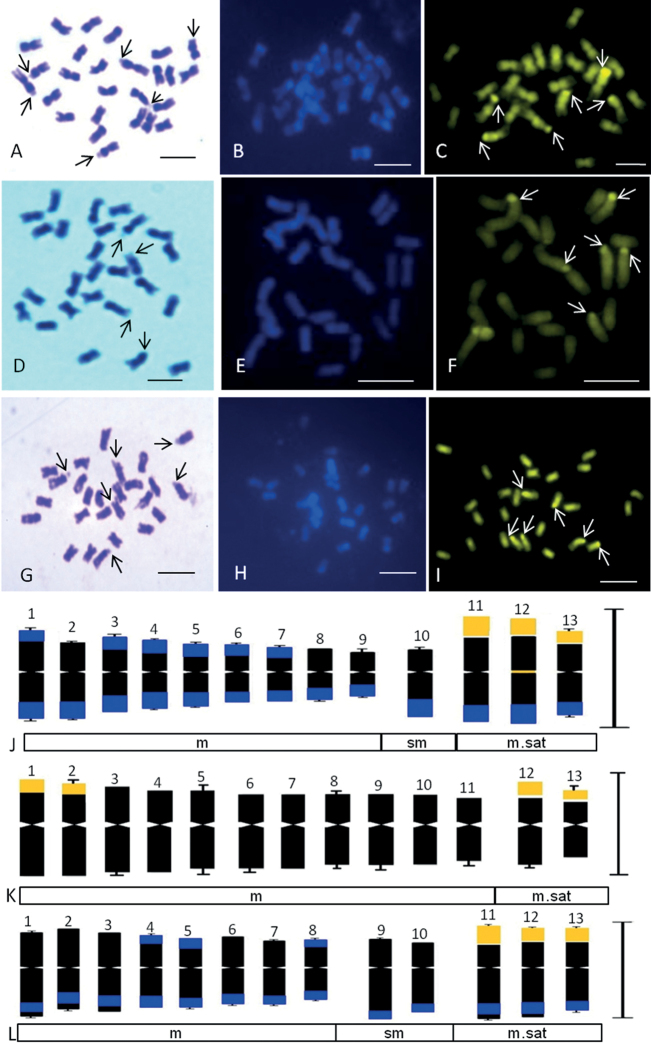
Somatic metaphase chromosomes and idiograms of *Luffa* species (2n = 26) stained with Giemsa (**A, D, G**), DAPI (**B, E, H**) and CMA3 (**C, F, I**) **A–C***L.acutangula***D–F***L.aegyptiacacylindrica***G–I***L.echinata*. Arrows indicate satellited chromosomes in Giemsa plates and CMA^+ve^ signals in **C, F, I**. Corresponding somatic idiograms (haploid set) of: **J***L.acutangula***K***L.aegyptiaca***L***L.echinata*, showing DAPI^+ve^ (blue) and CMA^+ve^ (golden yellow) bands. Scale Bars: 5 µm

**Figure 2.**
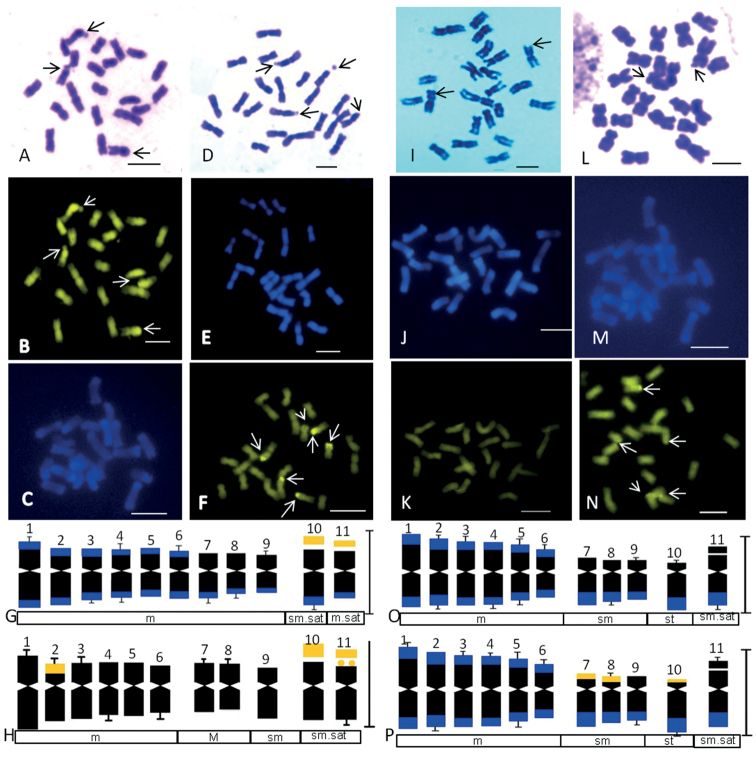
Somatic metaphase chromosomes and idiograms of *Trichosanthes* species stained with Giemsa (**A, D, I, L**), DAPI (**C, E, J, M**) and CMA3 (**B, F, K, N**) **A–C**T.cucumerinassp.cucumerina (2n = 22), **D–F**Trichosanthescucumerinassp.cucumerina ‘Anguina’ (2n = 22) **I–K***T.dioica* (male, 2n = 22) **L–N***T.dioica* (female, 2n = 22). Arrows indicate satellited chromosomes in Giemsa plates and CMA^+ve^ signals in **B, F, K, N**. Corresponding somatic idiograms (haploid set) of: **G**T.cucumerinassp.cucumerina**H**Trichosanthescucumerinassp.cucumerina ‘Anguina’ **O***T.dioica* male plant **P***T.dioica* female plant. Blue and golden yellow bands in idiograms indicate DAPI^+ve^ and CMA^+ve^ signals, respectively. Scale Bars: 5 µm

**Figure 3.**
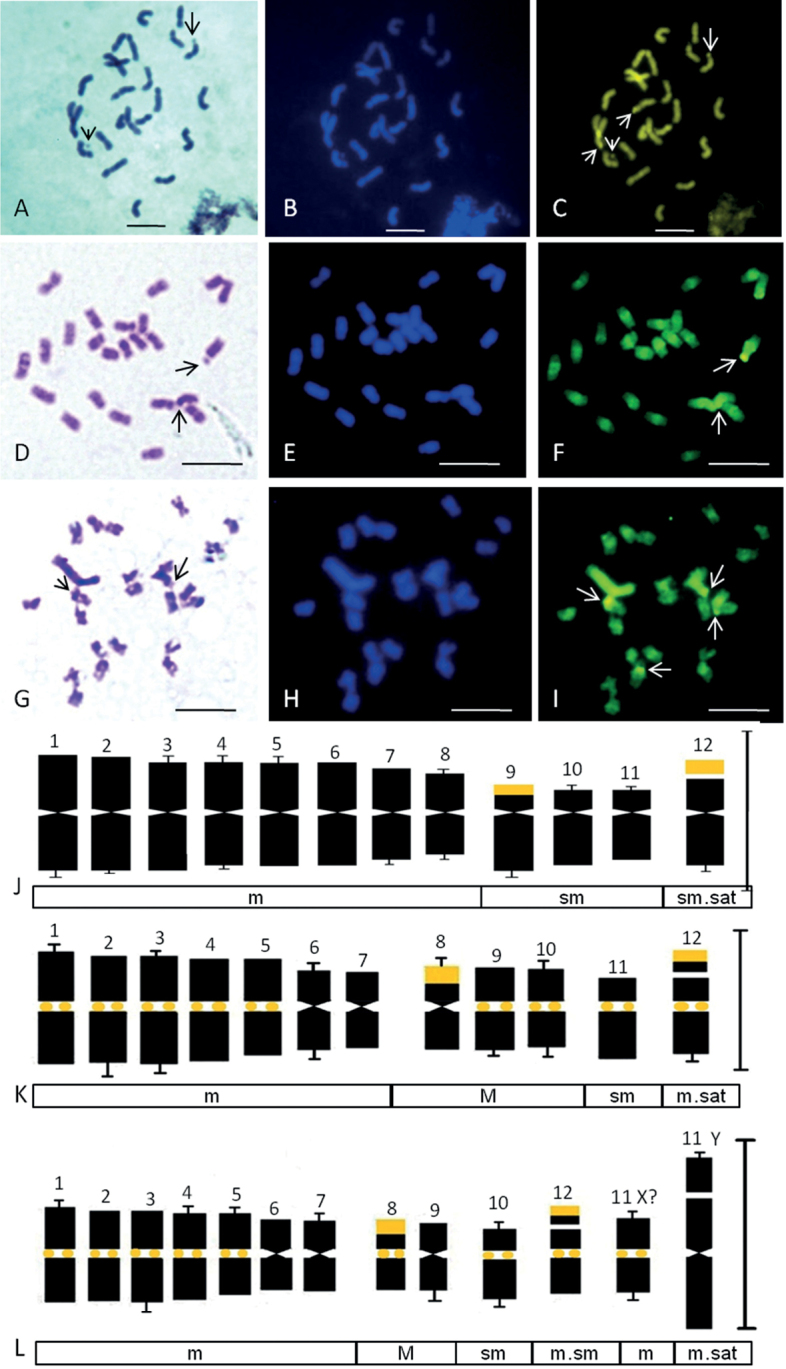
Somatic metaphase chromosomes and idiograms of two Benincaseae species (2n = 24) stained with Giemsa (**A, D, G**), DAPI (**B, E, H**) and CMA3 (**C, F, I**) **A–C***Benincasahispida***D–F***Cocciniagrandis* (female plant) **G–I***Cocciniagrandis* (male plant). Arrows indicate satellited chromosomes in Giemsa plates and distal CMA^+ve^ signals in **C, F, I**. Note the longest Y chromosome without any CMA band in **G–I** and centromeric CMA^+ve^ signals in **F, I**. Corresponding somatic idiograms (haploid set) of: **J***Benincasahispida***K***Cocciniagrandis* (female plant) **L***Cocciniagrandis* (male plant) with CMA^+ve^ (golden yellow) bands. Note the X chromosome remaining indistinguishable in **L**. Scale Bars: 5 µm

Along with the global review, fresh EMA based somatic plates and idiograms (Figs 1–3) of Indian species are presented here. We retain the previous designation of 10 tribes as ‘understudied’ (Bhowmick and Jha 2015b), excluding Indofevilleeae, having no cytological reports.

### ﻿Chromosome numbers

Currently, chromosome counts are available for 188 species (~19%) belonging to about 44 genera (~46%) of the 15 tribes, including the less attended ‘understudied tribes’. Within the ‘understudied tribes’, chromosome counts are available for only 42 species (out of almost 310) belonging to 17 genera (out of nearly 44). The basal number ranges from x/n = 5 (*Thladiantha* Bunge, 1833) to x/n = 15 (*Zanonia* Linnaeus, 1753) in these tribes (Table 2). Polyploidy has been abundantly reported in Gomphogyneae. Momordiceae have almost 60 species (Schaefer and Renner 2011) of which reports are known in nearly 11 species. The dibasic condition is noticed in *Momordica* Linnaeus, 1753 (x = 11 and 14) (Table 3) while polyploidy is detected in *M.charantia* Linnaeus, 1753 and *M.dioica* Willdenow, 1805 (2n = 56). *M.cymbalaria* Hooker, 1871, has the lowest count (2n = 18). In Bryonieae the X-Y sex determination system has been analysed in *Bryonia* Linnaeus, 1753 as the model along with *Ecballium* Richard, 1824 (Bhowmick and Jha 2015a). Chromosome counts are reported so far in 10 species of *Bryonia* (x = 10) and its sister genus *Ecballium* (x = 12 or x = 9, Table 4). Polyploidy is frequent in *Bryonia*. Sicyoeae is largest in terms of species (~264–266 species) (Schaefer and Renner 2011) of which cytological reports are known in around 14% species belonging to 9 genera (Table 5). Sicyoeae species range from x = 8 to x = 14 (Table 5). *Trichosanthes* Linnaeus, 1753 and *Luffa* Miller, 1754 have x = 11 and x = 13, respectively (Table 1). The less prevalent numbers include x = 12, x = 8 and x = 9 (Table 5). The possibility of multiple base number is noted in *Frantzia* Pittier, 1910 (x = 12/14) and *Sicyos* Linnaeus, 1753 (x = 12/13/14). Natural tetraploids are known in two species of *Trichosanthes* while the majority are diploids. Benincaseae is the second largest tribe comprising of 204–214 species in 24 genera (Schaefer and Renner 2011). Cytological reports are known in around 35% species (76 species of which 41 belong to *Cucumis* Linnaeus, 1753) of 12 genera (Tables 6, 7). x = 12 is the prevalent condition in Benincaseae (Tables 1, 6, 7). Dual base numbers are noted in the widely studied *Cucumis* (x = 7, 12). *Coccinia* Wight et Arnott, 1834 (x = 12) may also possess dual base numbers (x = 10 in *C.trilobata* Cogniaux, 1895). Molecular cytogenetics of *Cucumissativus* Linnaeus, 1753 has demonstrated the evolution of x = 7 from x = 12 in Benincaseae. x = 11 has been confirmed in *Citrullus* Schrader, 1836 and *Lagenaria*. The base number of *Melothria* Linnaeus, 1753, *Solena* Loureiro, 1790 and *Zehneria* Endlicher, 1833 can be x = 11 or x = 12 or both (Table 6). Cases of natural polyploidy are noted only in four species of *Cucumis* (Table 7). Cytogenetic information is available for 17 species in three genera of Cucurbiteae with x = 10 and many polyploids (Table 8). The zygotic chromosome numbers of *Luffa*, *Trichosanthes*, *Benincasa* Savi, 1818 and *Coccinia*, corroborate the previous reports (Figs 1–3, Table 1).

**Table 2.** Cytogenetic reports in the understudied tribes of Cucurbitaceae #.

Tribe and Genera	Species studied	Chromosome no.			Ploidy, Genome size, Chromosome features	References
		x	2n	n		
**Gomphogyneae***Gomphogyne* Griffith, 1845	*G.cissiformis* Griffith, 1837		32^a^	16^b^	Tetraploid^c^, autopolyploid^d^; 10 secondary constrictions, one pair satellited^e^; II, III, IV in meiosis^f^	CCDB^b^; Kumar and Subramaniam (1987)^a^, Singh (1990)^a,d^, Roy et al. (1991)^a,c,e,f^
*Hemsleya* F.B. Forbes et Hemsley, 1888	*H.amabilis* Diels, 1912, *H.carnosiflora* Wu et Chen, 1985, *H.chinensis* Forbes et Hemsley, 1888, *H.emeiensis* Shen et Chang, 1983, *H.graciliflora* Cogniaux, 1916, *H.heterosperma* Wallich, 1831, *H.macrocarpa* Cogniaux, 1916, *H.panacis*-*scandens* Wu et Chen, 1985, *H.sphaerocarpa* Kuang et Lu, 1982	7^a^	28^b^, 22^c^, 24^d^, 26^e^, 32^f^, 40^g^, 42^h^	14^h^	Tetraploid^i^, aneuploids^j^	Samuel et al. (1995)^a-j^, Anmin et al. (2011)^b, h^
*Gynostemma* Blume, 1825	*G.cardiospermum* Oliver, 1892	11^a^	66^b^		Hexaploid^c^	IPCN ^a-c^
	*G.guangxiense* Chen et Qin, 1988		22 ^a^		Diploid ^b^	IPCN ^a,b^
	*G.laxiflorum* Wu et Chen, 1983		22 ^a^		Diploid ^b^	IPCN ^a,b^
	*G.longipes* Wu et Chen, 1983		22^a^, 44^b^		Polyploid^c^	IPCN ^a-c^
	*G.microspermum* Wu et Chen, 1983		22^a^		Diploid ^b^	IPCN ^a,b^
	*G.pedatum* Blume, 1825	12^a^	24^b^		Diploid^c^	Roy et al. (1991) ^a,b,c^
	*G.pentagynum* Wang, 1989		22^a^		Diploid ^b^	IPCN ^a,b^
	*G.pentaphyllum* Thunberg, 1784		22^a^, 24^b^, 64^c^, 66^d^		Diploid^e^, triploid^f^, hexaploid^g^; 2C (flow cytometry): 3.62pg^h^; 17M+14sm+2st^i^; CSR: 2.16–4.09 µm^j^ 5S (8), 45S (10) rDNA and telomeric signals^k^	IPCN^a,b,c,e,f^; Zhang et al. (2013)^h^, Pellerin et al. (2018)^d,g,i,j,k^
	G.pentaphyllumvar.dasycarpum Wu, 1983		22^a^, 33^b^, 44^c^		Polyploid^d^	IPCN ^a-d^
	G.pentaphyllumvar.pentaphyllum Thunberg, 1784		22^a^, 44^b^, 66^c^, 88^d^		Polyploid^e^	IPCN ^a-e^
	*G.yixingense* Wang et Xie, 1981		88^a^		Polyploid^b^	IPCN ^a,b^
**Triceratieae**		8^a^			-	Roy et al. (1991) ^a^
*Fevillea* Linnaeus, 1753						
**Zanonieae***Zanonia* Linnaeus, 1753	*Z.indica* Linnaeus, 1759	15^a^	30^b^	15^c^	Autoploid^d^; Metacentric chromosomes^e^; CSR: 1.10-1.98 μm^f^	Lekhak et al. (2018) ^a-f^
**Actinostemmateae**	*A.lobatum* (Maxim.) Maxim. ex Franch. & Sav.		16^a^		-	IPCN ^a^
*Actinostemma* Griffith, 1841	*A.tenerum* Griffith, 1837		16^a^		Diploid^b^; 7M +1sm^c^; CSR: 2.88–4.02 µm^d^; 45S (1) rDNA and 45S+5S (1) rDNA adjacent signal^e^; telomeric repeat signals^f^	Pellerin et al. (2018) ^a-f^
**Thladiantheae**	*T.calcarata* Clarke, 1876, *T.cordifolia* Blume, 1826 *T.davidii* Franchet, 1886, *T.dentata* Cogniaux, 1916, *T.lijiangensis* Lu et Zhang, 1981, *T.nudiflora* Hemsley, 1887, *T.pustulata* Léveillé, 1916					
*Thladiantha* Bunge, 1833		3^a^, 5^b^, 9^c^	18^d^	5^e^, 9^f^	Diploid^g^	Darlington and Janaki Ammal (1945)^c^; Roy et al. (1991)^a,b,d,e,g^, IPCN^d,f^
	*T.dubia* Bunge, 1833		18^a^, 22^b^		Diploid^c^; 7M+1sm+1st^d^; CSR: 2.60–4.10 µm^e^; 45S (4) and co-localized 45S+5S (1) rDNA signals^f^; telomeric repeat signals^g^	Samuel et al. (1995)^b^, Pellerin et al. (2018)^a,c,d,e,f,g^
*Baijiania* Lu et Li, 1993	*B.yunnanensis* Lu et Zhang, 1984		32^a^		-	IPCN ^a^
**Siraitieae***Siraitia* Merrill, 1934	*S.grosvenorii* Swingle, 1941		28^a^		45S (6) and 5S (2) rDNA signals^b^	IPCN^a^, Li et al. (2007)^b^
**Joliffieae***Telfairia* Hooker, 1827	*T.occidentalis* Hooker, 1871		22^a^, 33^b^, 44^c^		Diploid^d^, aneuploid^e^, triploid^f^, Tetraploid^g^; 1 B^h^	Uguru and Onovo (2011) ^a-h^
	*T.pedata* Sims, 1826		22^a^		-	Bhowmick and Jha (2015)^a^
**Schizopeponeae***Herpetospermum* Hooker, 1867	*H.pedunculosum* Seringe, 1828			11^a^	45S (14), 5S (2) rDNA signals^b^	Xie et al. (2019a) ^a,b^
*Schizopepon* Maximowicz, 1859	*S.bryoniifolius* Maximowicz, 1859	10^a^	20^b^		-	Roy et al. (1991)^a^, IPCN^b^
**Coniandreae***Apodanthera* Arnott, 1841	*A.undulata* Gray, 1853	14^a^			-	IPCN ^a^
*Corallocarpus* Bentham et Hooker, 1867	*C.epigaeus* Rottler, 1803		26^a^	13^b^	-	Beevy and Kuriachan (1996) ^a,b^
	*C.welwitschii* Naudin, 1863		72^a^		-	Singh (1990)^a^
*Ibervillea* Greene, 1895		11^a^, 12^b^			-	Darlington and Janaki Ammal (1945) ^a,b^
*Kedrostis* Medikus, 1791	*K. africana* Linnaeus, 1753		40^a^		2C (feulgen densitometry): 0.8 pg^b^; 2C (flow cytometry): 1674 Mbp^c^	Bennet et al. (1982)^a,b^, Plant C DNA Values Database^c^
	*K.foetidissima* Jacquin, 1788		26^a^	13^b^		Beevy and Kuriachan (1996) ^a,b^
	*K.rostrata* Rottler, 1803	13^a^	26^b^	13^c^	-	IPCN ^a-c^
*Seyrigia* Keraudren, 1960				13^a^	-	IPCN ^a^

# x: base number; 2n: zygotic number; n: gametic number; CSR: chromosome size range; B: B chromosome; II: bivalents, III: trivalent, IV: tetravalent; superscripts correspond to references.

**Table 3.** Cytogenetic information in *Momordica* (Momordiceae)#.

Species	Chromosome no.		Ploidy, Genome size, Chromosome features	References
	2n	n		
*M.balsamina* Linnaeus, 1753	22^a^		Diploid^b^; two chromosomes with double constrictions^c^; CSR:0.65–1.98µm^d^; MCL:1.30µm^e^; TCL: 28.61µm^f^	Bharathi et al. (2011) ^a-f^
*M.charantia*	22^a^	11^b^	Diploid^e^, 2C (Feulgen densitometry): 4.10pg ^f^, 2C (flow cytometry): 1.43pg^g^; chromosomes mostly metacentric, few submetacentric and subtelocentric^h^; 2 chromosomes with satellites^i^; CSR: 1.26-1.81µm^j^; 45S (4) and 5S (2) rDNA signals^k^	Plant DNA C-Values Database^f^; Bharathi et al. (2011)^a,i^ ; Barow and Meister (2003)^g^; Lombello and Pinto-Maglio (2007)^a,b,h^; Bharathi et al. (2011); Waminal and Kim (2012)^a,e,h,j,k^; Kausar et al. (2015)^a^; Kido et al. (2016)^a,i^
M.charantiavar.charantia	22^a^	11^b^	Diploid^c^; 2C (flow cytometry): 0.72pg^d^; NORs: 4^e^; nucleolar and centromeric CMA^+^ bands^f^ ; CSR: 1.27-3.07µm^g^; MCL: 1.97 µm^h^; HCL: 21.77µm^i^	Ghosh et al. (2018)^a-f^; Ghosh et al. (2021)^a,c,d,g,h,i^
M.charantiavar.muricata Chakravarty, 1982	22^a^	11^b^	Diploid^c^; 2C (flow cytometry): 1.16pg^d^; NORs: 6^e^; nucleolar and centromeric CMA^+^ bands^f^; CSR: 1.64-3.13µm^g^; MCL: 2.19µm^h^; HCL: 24.19µm^i^	Ghosh et al. (2018)^a-f^; Ghosh et al. (2021)^a,c,d,g,h,i^
*M.cochinchinensis* Loureiro, 1790	28^a^	14^b^	Diploid^c^, 2C (flow cytometry): 2.64pg^d^, 6^e^ chromosomes with secondary constrictions; CSR:1.16–2.03µm^f^ /1.71-3.17μm^g^ ; MCL: 2.27µm^h^; HCL: 31.86μm^i^; 45S (8) and 5S (2) rDNA signals^j^	IPCN^b^; Xie et al. (2019a)^a,j^; Bharathi et al. (2011)^a,e,f^; Ghosh et al. (2021)^a,c,d,g,h,i^
*M.cymbalaria*	18^a^	8^b^, 9^c^, 11^d^	Diploid^e^, 2C (flow cytometry): 3.74 pg^f^; 2^g^–4^h^ chromosomes with secondary constrictions; CSR: 2.71-4.57μm^i^; MCL:3.75μm^j^; HCL: 33.79μm^k^	IPCN^b^; CCDB^b,d^; Bharathi et al. (2011)^a,c,e,g^; Ghosh et al. (2021)^a,e,f,h,i,j,k^
*M.denudata* Clarke, 1879		14^a^	-	IPCN ^a^
*M.dioica* Willdenow, 1805	28^a^, 56^b^		Diploid^c^; 2C (flow cytometry): 3.36 pg^d^, 2^e^-12^f^ chromosomes with secondary constrictions; CSR: 2.04-3.58μm^g^; MCL: 2.75µm^h^; HCL: 77.10μm^i^; 45S (4) and 5S (2) rDNA signals^j^	Bharathi et al. (2011)^a,c,e^; Xie et al. (2019a)^a,j^; Ghosh et al. (2021)^b,d,f,g,h,i^
*M.foetida* Schumacher, 1827	44^a^		-	Behera et al. (2011)^a^
*M.rostrata* Zimmermann, 1922	22^a^		-	Behera et al. (2011)^a^
*M.sahyadrica* Kattukunnel et Antony, 2007	28^a^		2 chromosomes with secondary constrictions^b^; CSR: 0.73–1.83µm^c^; TCL: 37.53µm^d^, MCL: 1.34µm^e^	Behera et al. (2011)^a-e^
*M.subangulata* Blume, 1826	56^a^		2C (flow cytometry): 3.06pg^b^; 8 chromosomes with secondary constrictions^c^; CSR: 1.52-3.11μm^d^; HCL: 60.30μm^e^	Ghosh et al. (2021) ^a-e^
M.subangulatasubsp.renigera Don, 1834	56^a^		4 chromosomes with secondary constrictions^b^; CSR: 0.52-1.26µm^c^; MCL: 0.93µm^d^; TCL 51.88µm^e^	Bharathi et al. (2011) ^a-e^
*M.tuberosa* Miquel, 1855	22^a^	11^b^	-	IPCN^a,b^; CCDB^a,b^

# 2n: Zygotic chromosome number; n: gametic chromosome number; I: univalent; II: bivalent; III: trivalent; CSR: chromosome size range; MCL: mean chromosome length; HCL: total length of haploid set of chromosomes; TCL: total length of diploid set of chromosomes; NOR: nucleolar organizing region, superscripts correspond to references.

**Table 4.** Chromosome number and genome size in Bryonieae#.

Genera	Species studied	Chromosome no.			Genome size	References
		x	2n	n		
*Bryonia*		10^a^				Darlington and Janaki Ammal (1945) ^a^
	*B.alba* Linnaeus, 1753	10^a^	20^b^	10^c^	2C (flow cytometry): 5827Mbp^d^	CCDB^d^ , Volz and Renner (2008)^a,b,c^
	*B.aspera* Ledebour, 1843	10^a^	40^b^, 60^c^	20^d^, 10^e^	-	Kumar and Subramaniam (1987)^c^, Volz and Renner (2008)^a,b,d,e^
	*B . cretica* Linnaeus, 1753	10^a^	60^b^	30^c^	-	Volz and Renner (2008) ^a,b,c^
	*B.dioica* Jacquin, 1774	10^a^	20^b^	10^c^	2C (microdensitometry): 4.01pg^d^; 2C (flow cytometry): 5522Mbp^e^	CCDB^d,e^, Volz and Renner (2008)^a,b,c^
	*B.macrostylis* Heilbronn et Bilge, 1954			10^a^	-	IPCN ^a^
	*B . marmorata* Petit, 1889		40^a^	20^b^	-	Volz and Renner (2008) ^a,b^
	*B . monoica* Aitchison et Hemsley, 1886	10^a^	20^b^		-	Volz and Renner (2008) ^a,b^
	*B.multiflora* Boissier et Heldreich, 1849	10^a^			-	Volz and Renner (2008) ^a^
	*B.syriaca* Boissier, 1856	10^a^	20^b^		-	Volz and Renner (2008) ^a,b^
	*B.verrucosa* Aiton, 1789	10^a^	20^b^	10^c^	2C (flow cytometry): 2.09pg^d^; 4504Mbp^e^	CCDB^d,e^, Volz and Renner (2008)^a–c^
*Ecballium*		12^a^				Darlington and Janaki Ammal (1945) ^a^
	*E.elaterium* Linnaeus, 1753		18^a^	12^b^	2C (flow cytometry): 2442Mbp^c^	Veselý (2012)^c^ , Volz and Renner (2008)^b^
	E.elateriumsubsp.dioicum Battandier, 1989		18^a^, 24^b^	9^c^, 12^d^	-	Volz and Renner (2008) ^a-d^
	E.elateriumLinnaeus, 1753subsp.elaterium		18^a^	9^b^	-	Volz and Renner (2008) ^a,b^

# 2n: Zygotic chromosome number; n: gametic chromosome number.

**Table 5.** Cytogenetic information in Sicyoeae#.

Genera studied	Species studied	Chromosome no.			Ploidy, Genome size, Chromosome features	References
		x	2n	n		
*Cyclanthera* Lilja, 1870		8^a^				Darlington and Janaki Ammal (1945) ^a^
	*C.pedata* (L.) Schrader, 1831		16^a^, 32^b^	8^c^	Diploid^e^	Roy et al. (1991)^a,c,e^, Samuel et al. (1995)^b^
*Echinocystis* Torrey et Gray, 1840		8^a^		16^b^		Bhowmick and Jha (2015b)^b^, Darlington and Janaki Ammal (1945)^a^
	*E.lobata* Michaux, 1803		16^a^, 32^b^		Tetraploid^c^; 2C (flow cytometry):1.49pg^d^	IPCN^a,b^, Plant DNA C-Values Database ^c,d^
	*E.macrocarpa* Greene, 1885		32^a^		-	Whitaker (1950) ^a^
*Echinopepon* Naudin, 1866	*E.wrightii* Gray, 1853			12^a^	-	IPCN^a^, CCDB^a^
*Frantzia*				12^a^, 14^b^	-	Schaefer and Renner (2011)^a,b^
*Hodgsonia* Persson, 1953	H.macrocarpavar.capniocarpa Ridley, 1920		18^a^		-	IPCN^a^, CCDB^a^
*Luffa*		13^a^				Darlington and Janaki Ammal (1945) ^a^
	*L.acutangula*	13^a^	26^b^	13^c^	Diploid^d^; CSR:1.39–3.20μm^e^; 18m+2sm+6m.st^f^; NORs:6^g^; distal DAPI and nucleolar CMA signals^h^	Kumar and Subramaniam (1987)^a,b^, IPCN^c^, Bhowmick and Jha (2021)^b,d-h^
	L.acutangulavar.acutangula			13^a^	-	Beevy and Kuriachan (1996) ^a^
	L.acutangulavar.amara Clarke, 1879			13^a^	-	Beevy and Kuriachan (1996) ^a^
	*L.aegyptiaca* (syn *L.cylindrica* Roemer, 1846)	13^a^	26^b^	13^c^	Diploid^d^, 2C (flow cytometry): 1.56 pg^e^; 2C (Feulgen densitometry): 1.7pg^f^; CSR: 1.60–2.06μm^g^; 24M+1sm^h^; 22m+ 4m.st^i^; NORs:2^j^; nucleolar and distal CMA signals^k^; 45S (10) and 5S (2) rDNA signals^l^	Bennet et al. (1982)^b,d,f^, Kumar and Subramaniam (1987)^a,b^, Waminal and Kim (2012)^b,d,g,h,l^, Bhowmick and Jha (2015a)^b,c,d,e,i,j,k^
	*L.echinata*		26^a^, 39^b^, 52^c^	13^d^	Diploid^e^; CSR 2.44–3.96 μm^f^; 16m+4sm+6m.st^g^; NORs: 6^h^; Distal and intercalary DAPI and nucleolar CMA signals^i^	Kumar and Subramaniam (1987)^a-e^, Bhowmick and Jha (2021)^a,e-i^
	*L.graveolens* Roxburgh, 1832	13^a^			-	Kumar and Subramaniam (1987) ^a^
	*L.hermaphrodita* Singh et Bhandari, 1963			13^a^	-	IPCN ^a^
	*L.operculata* Linnaeus, 1759	13^a^	26^b^	13^c^	-	Kumar and Subramaniam (1987)^a,b^, IPCN^c^
*Sicyos* (75, includes *Sechium*, *Microsechium*)		12^a^	24^b^		-	Darlington and Janaki Ammal (1945) ^a,b^
	*S.angulatus*	12^a^	24^b^		Diploid^c^; CSR: 1.9-4.6µm^d^; 4 adjacent 45S+5S rDNA signals^e^	Waminal and Kim (2015)^a-e^; IPCN^b^
	*S.australis* Endlicher, 1833		24^a^, 26^b^		12II^c^, 13II^d^	IPCN^a-d^, CCDB^a-d^
	*S.edulis* Jacquin, 1760 (syn of *Sechiumedule*)	13^a^	26^b^, 28^c^	12^d^, 13^e^	Diploid^f^; metacentric and submetaccentric chromosomes^g^; CSR: 2.69–5.38µm^h^; 45S (6), 5S (2) rDNA and telomeric repeat signals (28)^i^	Beevy and Kuriachan (1996)^a,b,e^, Pellerin et al. (2018)^c,f,g,h,i^, Ting et al. (2019)^c^, IPCN^d^, CCDB^d^
	*S.nihoaensis* St. John, 1970			12^a^	-	IPCN^a^, CCDB^a^
	*Sechiumcompositum* Smith, 1903 (syn. *Microsechiumcompositum*)			14^a^	-	IPCN^a^, CCDB^a^
	*S.hintonii* Wilson, 1958 (syn *Microsechiumhintonii*)			14^a^	-	IPCN^a^, CCDB^a^
*Trichosanthes* (100)		11^a^				Darlington and Janaki Ammal (1945) ^a^
	*T.anaimalaiensis* Beddome, 1864		22^a^	11^b^	-	Beevy and Kuriachan (1996) ^a,b^
	*T.boninensis* Nakai et Tuyama, 1928		22^a^		-	IPCN ^a^
	*T.bracteata* Lamarck, 1797	11^a^	22^b^, 44^c^, 66^d^		-	Kumar and Subramaniam (1987)^a,b^, Roy et al. (1991)^c,d^
	T.bracteatavar.bracteata			11^a^, 22^b^	-	Beevy and Kuriachan (1996) ^a,b^
	*T.chingiana* Handel-Mazzetti, 1936		22^a^		-	IPCN ^a^
	*T.costata* Blume, 1826 (syn *Gymnopetalumchinense* Loureiro, 1790)		22^a^		Diploid^b^; 45S (6) and 5S (4) rDNA signals^c^	Kumar and Subramaniam (1987)^a^, Xie et al. (2019a)^b,c^
	*T.cucumerina*		22^a^	11^b^	Diploid^c^; 12m+4M+2sm+4sm.st^d^; CSR: 2.26–4.99µm^e^; 6 chromosomes with double constrictions^f^; NORs: 4^g^; nucleolar CMA and distal DAPI bands^h^	Bhowmick and Jha (2019) ^a-h^
	T.cucumerinassp.cucumerina Anguina	11^a^	22^b^	11^c^, 22^d^, 32^e^, 33^f^	Diploid^e^; 2C (Feulgen densitometry): 2.2pg^f^; CSR: 2.77–5.01μm^g^; 12m+4M+2sm+4sm.st^h^; 6 chromosomes with double constrictions^i^; NORs: 4^j^; nucleolar and distal CMA bands^k^; 45S (6) and 5S (2) rDNA signals^l^	Kumar and Subramaniam (1987)^a^, Bhowmick and Jha (2019)^b,c,e,g,h,i,j,k^, Xie et al. (2019a)^b,l^, IPCN^c-f^
	*T.dioica*	11^a^	22^b^	11^c^	Diploid^d^; 2C (flow cytometry): male-2.27pg, female- 2.32 pg^e^; 12m+6Sm +2St+2Sm.t^f^; distal DAPI bands^g^; distal CMA bands in females^h^; 1 rod bivalent in meiosis^i^	Kumar and Subramaniam (1987)^a^, Guha et al. (2004)^b,d,f,h^, Bhowmick and Jha (2015a)^b,c,d,e,f,g,h,i^
	*T.dunniana* Léveillé, 1911		22^a^		Diploid^b^; 45S (6) and 5S (2) rDNA signals^c^	Xie et al. (2019a) ^a-c^
	*T.himalensis* Clarke, 1879			11^a^	-	Roy et al. (1991) ^a^
	*T.hupehensis* Cheng et Yueh, 1974		22^a^		-	IPCN^a^, CCDB^a^
	*T.kirilowii* Maximowicz, 1859		60^a^, 66^b^, 88^c^, 110^d^		Hexa-, octa-, decaploid^e^;CSR: 2.3-3.5μm^f^; 45S (4), 5S (4) and 45S +5S (6) adjacent rDNA signals^g^	IPCN^a^, CCDB^a^, Waminal and Kim (2015)^b,c,d,e-g^
	T.kirilowiivar.japonica			11^a^	-	Roy and Saran (1990)^a^
	*T.lepiniana* Naudin, 1868		44^a^	11^b^	1 B^c^	Roy et al. (1991)^b,c^, IPCN^a^, CCDB^a^
	*T.lobata* Roxburgh, 1832	11^a^		11^b^	-	Kumar and Subramaniam (1987)^a^, Beevy and Kuriachan (1996)^b^
	*T.mianyangensis* Yueh et. Liao, 1992		88^a^		-	IPCN^a^, CCDB^a^
	*T.nervifolia* Linnaeus, 1753			11^a^	-	Beevy and Kuriachan (1996) ^a^
	*T.ovigera* Blume, 1826		22^a^		Diploid^b^; 45S (10) and 5S (2) rDNA signals^c^	Xie et al. (2019a) ^a-c^
	*T.palmata* Linnaeus, 1759		22^a^, 44^b^, 66^c^	11^d^		IPCN ^a-d^
	*T.pedata* Merril et Chun, 1934		22^a^		-	IPCN^a^, CCDB^a^
	*T.truncata* Clarke, 1879		22^a^		-	IPCN^a^, CCDB^a^
	*T.wallichiana* Wight, 1840	11^a^	22^b^		-	Kumar and Subramaniam (1987) ^a,b^

#x: base number; 2n: zygotic number; n: gametic number; NOR: nucleolar organizing region; B: B chromosome; II: bivalents; superscripts correspond to references.

**Table 6.** Cytogenetic information on Benincaseae#.

Genera studied	Species studied	Chromosome no.			Ploidy, Genome size, Chromosome features	References
		x	2n	n		
*Benincasa*	*B.fistulosa*		24^a^		Diploid^b^; 45S (4) and 5S (4) signals^c^	Li et al. (2016) ^a,b,c^
	*B.hispida*	12^a^	24^b^	12^c^	Diploid^d^; 2C (flow cytometry):1.97pg^e^, 2C (feulgen densitometry):2.1pg^f^; CSR 2.54-4.59µm^g^; 16m+6Sm+2Sm.t^h^; NORs:2^i^; distal CMA signals^j^; 45S (2) and 45S+5S (2) adjacent rDNA signals^k^	Plant DNA C-Values Database^f^, Waminal et al. (2011)^b,d,g,k^, Bhowmick and Jha (2015a)^b,c,d,e,h,i,j^
*Citrullus*		11^a^				Darlington and Janaki Ammal (1945) ^a^
	*C.amarus* (syn. C.lanatusvar.citroides)	11^a^	22^b^		Diploid^c^; CSR: 3.1–4.7μm^d^; 45S (2) and 5S (4) rDNA signals^e^	Reddy et al. (2013)^b-d^, Waminal and Kim (2015)^a-e^, Renner et al. (2017)^b^
	*C.colocynthis*		22^a^	11^b^	Diploid^c^; 45S (2) and 45S+5S (2) adjacent rDNA signals^d^	Beevy and Kuriachan (1996)^b^, Reddy et al. (2013)^a,c,d^, Li et al. (2016)^a,c,d^
	*C.ecirrhosus*		22^a^	11^b^	Diploid^c^; 2 satellites detected in meiosis^d^; 45S (2) and 5S (4) rDNA signals^e^; regular meiosis^f^	Li et al. (2016)^a,c,e^, Renner et al. (2017)^a,b,c,d,f^
	*C.lanatus*		22^a^	11^b^	Diploid^c^; CSR: 1.09μm-1.72μm^d^; 14m+8sm^e^; 45S (2) and 45S+5S (2) adjacent rDNA signals^f^; linkage groups hybridized to chromosomes^g^	Beevy and Kuriachan (1996)^b^, Waminal et al. (2011)^a,c,d,e,f^, Ren et al. (2012)^a,c,g^
	C.lanatussubsp.lanatus		22^a^		Diploid^b^; 45S (2) and 5S (4) rDNA signals^c^	Li et al. (2016) ^a-c^
	C.lanatussubsp.mucosospermus Fursa, 1972		22^a^		Diploid^b^; 45S (2) and 45S+5S (2) adjacent rDNA signals^c^	Li et al. (2016) ^a-c^
	C.lanatussubsp.vulgaris Schrader, 1836		22^a^		Diploid^b^; 45S (2) and 45S+5S (2) adjacent rDNA signals^c^	Li et al. (2016) ^a-c^
	C.lanatusvar.lanatus		22^a^		Diploid^b^; 45S (2) and 45S+5S (2) adjacent rDNA signals^c^	Reddy et al. (2013) ^a-c^
	*C.naudinianus* (syn *Acanthosicyosnaudinianus*)		24^a^		Diploid^b^; 45S (2) and co-localized 45S+5S (2) rDNA signals^c^	Li et al. (2016) ^a,b,c^
	*C.rehmii*		22^a^		Diploid^b^; 45S (2) and 5S (2) rDNA signals^c^	Reddy et al. (2013)^a-c^, Li et al. (2016)^a-c^
	*C.vulgaris* Schrader, 1836		22^a^, 44^b^	11^c^	Diploid^d^; 2C: 0.88/0.90pg^e^	IPCN^a,c,d^, Arumuganathan and Earle (1991)^e^
*Coccinia* (30)		12^a^				Darlington and Janaki Ammal (1945) ^a^
	*C.abyssinica* Lamarck, 1753	12^a^	24^b^		-	Kumar and Subramaniam (1987)^a^, Roy et al. (1991)^b^
	*C.grandis*	12^a^	24^b^	12^c^	Diploid^d^, 2C (Flow cytometry): male- 0.943^e^/0.92^f^ pg and female- 0.849^g^/ 0.73^h^ pg; CSR: 1.33-4.71μm (male) and 1.35-2.26µm (female)^i^; 15m+4M+2sm+2m:sm+1m:st (Y) in male and 14m+6M+2sm+2m:st in female^j^; NORs-2^k^; chromosomal C bands^l^; centromeric, nucleolar CMA bands^m^; 45S (4)^n^ rDNA signals, 2 signals adjacent to 5S^o^; GISH performed^p^; repetitive, organellar DNA hybridized^q^; centromere immunofluorescence^r^; heteromorphic sex chromosomes (largest Y)^s^; X-Y bivalent (meiosis)^t^	Bhowmick et al. (2012)^b,c,d,j,k,m,s,t^, (2016) ^b,d,f,h,j,k,n,s^, Sousa et al. (2013)^b,d,e,g,i,k,l,n,o,r,s,t^, Sousa et al. (2017)^b,d,k,n,o,p,q,r,s^, Xie et al. (2019a)^b,n,o^
	*C.hirtella* Cogniaux, 1896		24^a^		Diploid^b^; 2C (flow cytometry): male-0.988pg^c^; 45S (4) and 45S+5S (2) adjacent rDNA signals^d^, repetitive and organellar DNA hybridized^e^; centromere immunofluorescence performed^f^	Sousa et al. (2017) ^a-f^
	*C.sessilifolia* Sonder, 1881		24^a^		Diploid^b^; 2C (Flow cytometry): male- 0.984pg, female- 0.998pg^c^; 45S (4) and 45S+5S (2) adjacent rDNA signals^d^; repetitive and organellar DNA^e^; centromere immunofluorescence performed^f^	Li et al. (2016)^a,b,d^, Sousa et al. (2017)^a–f^
	*C.trilobata*		20^a^		Diploid^b^; 2C (flow cytometry): male- 1.263pg ^c^; 45S (2) and 45S+5S (2) adjacent rDNA signals^d^, repetitive, organellar DNA sequence hybridized^e^	Sousa et al. (2017) ^a-e^
*Ctenolepis* Hooker, 1867	*C.garcinii* Burman, 1768		24^a^	12^b^	-	Kumar and Subramaniam (1987)^a^, Beevy and Kuriachan (1996)^b^
*Diplocyclos* Endlicher, 1833	*D.palmatus*		24^a^		Diploid^b^; 45S (4) and 45S+5S (2) adjacent rDNA signals^c^	Li et al. (2016) ^a-c^
*Lagenaria* Seringe, 1825	*L.leucantha* Rusby, 1896		22^a^	11^b^	-	IPCN^a,b^, CCDB^a,b^
	L.leucanthavar.clavata Makino, 1940		22^a^		-	CCDB ^a^
	*L.siceraria*	11^a^	22^b^	11^c^	Diploid^d^, 2C (flow cytometry): 0.734pg^e^; 2C (Feulgen densitometry):1.4pg^f^; CSR: 0.56–1.06μm^g^; metacentric and few sub-metacentric chromosomes^h^; 45S (2) and 45S+5S (2) adjacent rDNA signals^i^	Darlington and Janaki Ammal (1945)^a^, Plant DNA C-Values Database^f^, Beevy and Kuriachan (1996)^c^, Achigan-Dako et al. (2008)^d,e^, Waminal and Kim (2012)^b,d,g,h,i^, Li et al. (2016)^b,d,i^, Xie et al. (2019a)^b,i^
	L.sicerariavar.macrocarpa		22^a^		-	CCDB ^a^
	*L.vulgaris* Seringe, 1825		22^a^	11^b^	Diploid^c^; 2C (Feulgen densitometry): 1.40pg^d^	Bennet et al. (1982)^a,b,c^
*Melothria*		11^a^, 12^b^				Darlington and Janaki Ammal (1945) ^a,b^
	*M.pendula* Linnaeus, 1753		24^a^		Diploid^b^; 45S (2) and 45S+5S (2) adjacent rDNA signals^c^	Li et al. (2016) ^a-c^
	*M.perpusilla* Blume, 1826		48^a^		-	Kumar and Subramaniam (1987) ^a^
	*M.scabra* Naudin, 1866		24^a^		-	CCDB ^a^
*Peponium* Engler, 1897	*P.betsiliense* Keraudren, 1960		24^a^		-	CCDB ^a^
*Solena*	*S.amplexicaulis* Lamarck, 1785 (syn. *S.heterophylla*, *Melothriaheterophylla*, *Zehneriaumbellata*)		22^a^, 24^b^, 26^c^, 36^d^, 48^e^	11^f^, 12^g^, 24^h^	2-4 B^i^	Kumar and Subramaniam (1987)^a,b,c,e^, Roy et al. (1991)^d,i^, Beevy and Kuriachan (1996)^b,g,h^, IPCN^b,d,e,f,g,h^
*Zehneria*	*Z.capillacea* Jeffrey, 1962 (syn. *Melothriacapillacea*)		22^a^		-	CCDB ^a^
	*Z.indica* Loureiro, 1790 (syn. *Melothriajaponica*)	11^a^	22^b^	24^c^	Diploid^d^; 45S (2) and 45S+5S (2) adjacent rDNA signals^e^	Waminal and Kim (2015) ^a,b,d,e^
	*Z.marlothii* Cogniaux, 1962		24^a^		Diploid^b^; 45S (2) and 45S+5S (2) adjacent rDNA signals^c^	Li et al. (2016) ^a,b,c^
	*Z.maysorensis* Wight et Arnott, 1834		48^a^	24^b^	45S (2) and 5S (2) signals^c^	Beevy and Kuriachan (1996)^a,b^, Xie et al. (2019a)^a,c^
	*Z.mucronata* Blume, 1856 (syn. *Melothriamucronata*)		22^a^	12^b^	-	Darlington et al. (1956)^a^, CCDB^b^
	*Z.scabra* Sonder, 1862 (syn. *Melothriapunctata*)		24^a^, 48^b^		-	CCDB^a^, Kumar and Subramaniam (1987)^b^
	*Z.thwaitesii* Schweinfurth, 1868		44^a^		-	CCDB ^a^

# genera included other than *Cucumis*; x: base number; 2n: zygotic number; n: gametic number; NOR: nucleolar organizing region; B: B chromosome; II: bivalents; IPCN: Index to Plant Chromosome Number Reports; CCDB: Chromosome Counts Database; superscripts correspond to references.

**Table 7.** Cytogenetic features of *Cucumis* (Benincaseae)#.

Species with subspecies/ varieties				Chromosome no.			Ploidy, Genome size, Chromosome features	References
				x	2n	n		
*C.aculeatus* Cogniaux, 1895					48^a^		Allotetraploid^b^; 24II^c^	IPCN^a-c^, CCDB^a-c^
*C.africanus* Linnaeus, 1782				12^a^	24^b^, 48^c^	12^d^	Diploid^e^; 2C (Feulgen microdensitometry): 1.782pg^f^; 4 satellited chromosomes^g^; 45S (4^h^/6^i^) rDNA signals, 2 co-localized 45S+5S signals^j^	IPCN^b,c^, Yadava et al. (1984)^b,d^, Ramachandran and Narayan (1985)^b,e,f^, Yagi et al. (2015)^a,b,g,i,j^, Zhang et al. (2016)^b,e,h,j^
*C.angolensis* Cogniaux, 1881.					24^a^			IPCN ^a^
*C.anguria* Linnaeus, 1753					24^a^		Diploid^b^; majorly submetacentric and few nearly metacentric chromosomes^c^; 1 pair satellited^d^; 45S (2) and co-localized 45S+5S (2) rDNA signals^e^, ScgCP enables chromosome identification^f^; GISH reveals cross species relationships^g^	Singh and Roy (1974)^a-d^, Zhang et al. (2015)^a,g^, (2016)^b,e^, Li et al. (2018)^a,f^
			C.anguriavar.anguria	12^a^	24^b^	12^c^	Diploid^d^; 4 satellited chromosomes^e^; 45S (2) and co-localized 45S+5S (2) rDNA signals^f^	Yadava et al. (1984)^b,c^, Yagi et al. (2015)^a,d,e,f^
			C.anguriavar.longipes		24^a^	12^b^	Diploid^c^; 2C (Feulgen microdensitometry): 1.587pg^d^	Yadava et al. (1984)^a,b^, Ramachandran and Narayan (1985)^a,c,d^
			C.anguriavar.longaculeatus	12^a^	^12.5^		Diploid^c^; 4 satellited chromosomes^d^; 45S (2) and co-localized 45S+5S (2) rDNA signals^e^	Yagi et al. (2015) ^a-e^
*C.asper* Cogniaux, 1901					24^a^		Diploid^b^; 45S (4) and 5S (2) signals detected^c^	IPCN^a^, Zhang et al. (2016)^a,b,c^
*C.callosus* Rottler, 1803					14^a^, 24^b^	12^c^	Diploid^d^; 2C (Feulgen microdensitometry):1.590pg^e^; 11m+1sm (haploid)^f^	Ramachandran and Narayan (1985)^a,d,e^, Rajkumari et al. (2013)^b,c^, (2015)^b,c,f^
*C.cinereus*Cogniaux, 1901 (syn. *Cucumellacinerea*)							2C (Feulgen microdensitometry): 0.5pg^a^	Bennet et al. (1982)^a^
*C.diniae* Raamsdonk et Visser, 1992					48^a^		-	IPCN ^a^
*C.dinteri* Cogniaux, 1901					24^a^		Diploid^b^; 2C (Feulgen microdensitometry): 2.167pg^c^	IPCN^a^, Ramachandran and Narayan (1985)^a-c^
*C.dipsaceus* Spach, 1838					24^a^	12^b^	Diploid^c^; 2C (Feulgen microdensitometry): 2.448pg^d^; 2m+8sm+2st (haploid)^e^; 45S (2) and co-localized 45S+5S (2) rDNA signals^f^	Yadava et al. (1984)^a,b^, Ramachandran and Narayan (1985)^a,c,d^, Rajkumari et al. (2015)^a,c,e^, Zhang et al. (2016)^a,c,f^
*C.ficifolius*Richard, 1847					24^a^, 48^b^	12^c^	Diploid^d^; 2C (Feulgen microdensitometry):1.373pg^e^; 45S (2) and co-localized 45S+5S (2) rDNA signals^f^	Yadava et al. (1984)^a,c^, Ramachandran and Narayan (1985)^a,d,e^, Zhang et al. (2016)^b,f^
*C.figarei* Naudin, 1859					48^a^, 72^b^		Autoallopolyploid^c^; 2C (Feulgen microdensitometry): 3.886pg^e^; 36II^f^	IPCN^a-f^, Ramachandran and Narayan (1985)^a,c,e^
*C.heptadactylis* Naudin, 1859					48^a^	23^b^, 24^c^, 52^d^	Autotetraploid^e^; 2C (Feulgen microdensitometry): 2.225pg^f^; 8 satellited chromosomes^g^; 45S (8) rDNA signals^h^ of which 4 co-localized to 5S signals^i^ or separate 5S (4) rDNA signals^j^; 10IV+4II^k^; irregular meiosis^l^	IPCN^a,e,k^, Yadava et al. (1984)^a,b,d,e,l^, Ramachandran and Narayan (1985)^a,e,f^, Yagi et al. (2015)^a,e,g,h,i^, Zhang et al. (2016)^a,e,h,j^
*C.hookeri* Naudin, 1870					24^a^	12^b^	Diploid^c^	Yadava et al. (1984) ^a,b,c^
*C.humifructus* Stent, 1927					24^a^		Diploid^b^; 2C (Feulgen microdensitometry): 2.455 pg^c^	Ramachandran and Narayan (1985) ^a,b,c^
*C.hystrix* Chakravarty, 1952				12^a^	24^b^		Diploid^c^; 2m+10sm (haploid)^d^; 45S (4) and co-localized 45S+5S (2) rDNA signals^e^; FISH with bulked oligo probe from cucumber chromosome C7^f^, GISH reveals cross species relationships^g^	Rajkumari et al. (2015)^b,c,d^, Han et al. (2015)^f^, Zhang et al. (2015)^b,g^, (2016)^a,b,c,e^
*C.indicus* Ghebretinsae et Thulin, 2007					20^a^		Diploid^b^; 4m+ 6sm (haploid)^c^	Rajkumari et al. (2015) ^a-c^
*C.javanicus* Miquel, 1856 (syn. *Melothriaassamica*)				12^a^	24^b^, 48^c^		-	Kumar and Subramaniam (1987)^a^, CCDB^b,c^
*C.leiospermus* Wight et Arnott, 1834 (syn. *Melothrialeiosperma*)					24^a^		-	CCDB ^a^
*C.leptodermis* Schweickerdt, 1933					24^a^	12^b^	-	Yadava et al. (1984) ^a,b^
*C.longipes* Hooker, 1871					24^a^		-	IPCN ^a^
*C.meeusei* Jeffrey, 1965					48^b^	22^c^, 24^d^	Tetraploid^e^; 2C- 3.203pg (Feulgen microdensitometry)^f^; 45S (6) and co-localized 45S+5S (2) rDNA signals^g^	Yadava et al. (1984)^b,c,d^, Ramachandran and Narayan (1985)^b,e,f^, Zhang et al. (2016)^b,e,g^
*C.melo* Linnaeus, 1753				12^a^	20^b^, 22^c^, 24^d^	12^e^	Diploid^f^; 2C (Feulgen photometry) : 0.94-1.04pg^g^, 1.90pg^h^; 2C (Flow cytometry): 1.05pg^i^; 14m+10st (2SAT)^j^; 7m+5sm (haploid)^k^; 4 satellites^l^ or 2 satellites^m^; CSR1.0-2.1μm^n^; CMA bands detected^o^; 45S (2) and co-localized 45S+5S (2) rDNA signals^p^; centromeric, telomeric, nulceolar and SSR probe hybridization reveals chromosomal relation^q^; ScgCP applied for comparative chromosome rearrangement studywith *C.sativus*^r^; FISH with bulked oligo probe from cucumber chromosome C7^s^; novel centromeric satellite DNA hybridized on chromosomes^t^; GISH reveals cross species relationships^u^; infraspecific positional differences in 45S (terminal and interstitial) -5S (terminal, subterminal and interstitial) rDNA signals^v^	CCDB^b,c^, Plant DNA C-Values Database^h^, Kumar and Subramaniam (1987)^a^, Arumuganathan and Earle (1991)^g^, Marie and Brown (1993)^i^, Zhang (2005)^d,f,j,u^, (2015)^d,t^, Song and Kim (2008)^d,f,m^, Han et al. (2009)^d,e,f,q^, (2015)^s^, Liu et al. (2010)^d,f,q^, Hoshi et al. (2013)^d,f,l,n,o,p^, Lou et al. (2014)^r^, Rajkumari et al. (2013)^d,e^, (2015)^d,f,k^, Setiawan et al. (2018)^d,v^, (2020)^d,t^
			C.melosubsp.melo	12^a^	24^b^		Diploid; 45S (4) and 5S (2) rDNA signals^c^	Zhang et al. (2016) ^a-c^
			C.melosubsp.agrestis Naudin, 1859	12^a^	24^b^		Diploid; 45S (4) and 5S (2) rDNA signals^c^	Zhang et al. (2016) ^a-c^
			C.melovar.agrestis	12^a^	24^b^	12^c^	Diploid^d^; 2C (Feulgen microdensitometry): 2.483pg^e^; 10m+2sm (haploid)^f^; 1 pair satellited^g^	Singh and Roy (1974)^b,d,g^, Yadava et al. (1984)^a-d^, Ramachandran and Narayan (1985)^b,d,e^, Beevy and Kuriachan (1996)^b,c^, Rajkumari et al. (2015)^b,d,f^
			C.melovar.conomon Thunberg, 1780		24^a^		Diploid^b^; 7m+3sm+2st (haploid)^c^	Zhang et al. (2005)^a^, Rajkumari et al. (2015)^a,b,c^
			C.melovar.flexuosus Linnaeus, 1763		24^a^		-	IPCN ^a^
			C.melovar.inodorus Jacquin, 1832		24^a^		Diploid^b^; 2C (flow cytometry): 0.64pg^c^	Karimzadeh et al. (2010) ^a-c^
			C.melovar.melo		24^a^	12^b^	Diploid^c^; 4m+8sm (haploid)^d^	Beevy and Kuriachan (1996)^a,b^, Rajkumari et al. (2015)^b,c,d^
			C.melovar.momordica Roxburgh, 1832		24^a^	12^b^	Diploid^c^; 2C (Feulgen microdensitometry): 2.291pg^d^; 6m+5sm+1st (haploid)^e^	Yadava et al. (1984)^a,b^, Ramachandran and Narayan (1985)^a,c,d^, Rajkumari et al. (2015)^a,c,e^
			C.melovar.muskmelon		24^a^	12^b^		Yadava et al. (1984) ^a,b^
			C.melovar.utilissimus Roxburgh, 1832		24^a^	12^b^	Diploid^c^; 2C (Feulgen densitometry): 2.358 pg^d^	Yadava et al. (1984)^a,b^; Ramachandran and Narayan (1985)^a,c,d^
*C.membranifolius* Hooker, 1871					48^a^	24^b^	-	Yadava et al. (1984) ^a,b^
*C.metulifer* Naudin, 1859 (syn. *C.metuliferus*)					24^a^	12^b^	Diploid^c^; 2C (Feulgen microdensitometry): 2.391pg^d^; metacentric, submetacentric, subtelocentric chromosomes^e^; CSR: 0.9–2.0 μmf; 4 satellites^g^; nucleolar and centromeric CMA-DAPI bands^h^; 45S (2) and co-localized 45S+5S (2) rDNA signals^i^, satellite sequences^j^ and telomeric DNA^k^ hybridized on chromosomes; ScgCP applied for comparative chromosome rearrangement studywith *C.sativus*^l^; GISH reveals cross species relationships^m^	Yadava et al. (1984)^a,b,c^, Ramachandran and Narayan (1985)^a,c,g^, Ramachandran and Narayan (1990)^a,c,i^, Hoshi et al. (2013)^a,c,e,f,g,h^, Lou et al. (2014)^l^, Yagi et al. (2014)^a,c,g,h,i,j,k^, Li et al. (2016)^a,c,i^, Zhang et al. (2015)^a,m^, (2016)^a,c,i^
*C.myriocarpus*					24^a^	12^b^	Diploid^c^; 45S (2^d^/4^e^) and co-localized 45S+5S (2)^f^ rDNA signals^d^	CCDB^a^; Zhang et al. (2016)^a,b,c,d,f^, Yagi et al. (2015)^a-f^
			C.myriocarpussubsp.leptodermis Schweickerdt, 1933	12^a^	24^b^		Diploid^c^; 4 satellited chromosomes^d^; 45S (3^e^, 2^f^) and co-localized 45S+5S (2^g^) rDNA signals	Yagi et al. (2015) ^a-g^
			C.myriocarpusvar.myriocarpus	12^a^	48^b^		Tetraploid^c^; 8 satellited chromosomes^d^; 45S (4) and co-localized 45S+5S (4) rDNA signals^e^	Yagi et al. (2015) ^a-e^
*C.prophetarum* Linnaeus, 1755					24^a^	12^b^	Diploid^c^, 2C (Feulgen Microdensitometry): 1.656 pg^d^ 5m+7sm (haploid)^e^	Ramachandran and Narayan (1985)^a,c,d^, Rajkumari et al. (2013)^a,b^, (2015)^a,c,e^
	C.prophetarumsubsp.zeyheri Sonder, 1862				48^a^		-	IPCN ^a^
*C.pubescens* Willdenow, 1805					24^a^	12^b^	-	IPCN^a^; Beevy and Kuriachan (1996)^b^
*C.pustulatus* Hooker, 1871					48^a^, 72^b^	24^c^	Hexaploid^d^, 45S (8) and co-localized 45S+5S (2) rDNA signals^e^; FISH with bulked oligo probe from cucumber chromosome C7^f^	Yadava et al. (1984)^a,c^, Han et al. (2015)^f^, Zhang et al. (2016)^b,d,e^
*C.ritchiei* Clarke, 1879					24^a^		Diploid^b^, 8m+4sm (haploid)^c^	Rajkumari et al. (2015) ^a,b,c^
*C.sagittatus* Peyritsch, 1860					24^a^	12^b^	Diploid^c^, 2C (Feulgen microdensitometry):1.571pg^d^	Yadava et al. (1984)^a,b^, Ramachandran and Narayan (1985)^a,c,d^
*C.sativus* Linnaeus, 1753				7^a^	14^b^	7^c^	Diploid^d^, 2C (flow cytometry): 1.03pg^e^ /1.77pg^f^; 12 metacentric and 2 sub-metacentric chromosomes^g^; CSR: 0.83-1.01μm^h^, chromosomal C-bands^i^; centromeric 45S (10) and distal 5S (2) rDNA signals^j^; FISH with centromeric and telomeric^k^ and SSR probe reveals chromosome evolution^l^; high resolution molecular cytogenetic map^m^; ScgCP applied for cross species chromosome rearrangement study^n^; FISH with bulked oligo probe from cucumber chromosome C7 in comparison with 5 *Cucumis* species^o^; GISH reveals cross species relationships^p^	Kumar and Subramaniam (1987)^a,b^, Marie and Brown (1993)^f^, Beevy and Kuriachan (1996)^b,c^, Hoshi et al.(2008)^b,d,i^, Barow and Meister (2003)^b,d,e^, Han et al (2011)^b,d,k,l,m^, Liu et al. (2010)^b,l^, Waminal and Kim (2012)^b,d,g,h,j^, Rajkumari et al. (2013)^b,c,f^, Sun et al. (2013)^b,m^, Lou et al. (2014)^b,n^, Han et al. (2015)^o^, Zhang et al. (2015)^b,p^, Li et al. (2016)^b,d,j^
		*C.sativus* var. Hokutosei		7^a^	14^b^		Diploid^c^, 12 metacentric, 2 sub-metacentric chromosomes^d^; centromeric and telomeric signals^e^	Zhang et al. (2012) ^a-e^
		C.sativusvar.hardwickii Royle, 1835		7^a^	14^b^		Diploid^c^; 2C (Feulgen Microdensitometry): 1.798pg^d^; 6m+1sm (haploid)^e^; centromeric 45S (6) and intercalary 5S (2) rDNA signals^f^, centromeric, telomeric and SSR probe hybridization^gh^; molecular cytogenetic map^i^	Ramachandran and Narayan (1985)^b,d^, Zhao et al. (2011)^b,c,f,g^, Yang et al. (2012)^b,h^, Rajkumari et al. (2015)^b,c,e^, Zhang et al. (2016)^a,b,c,f^

		*C.sativus* var. Long green		7^a^	14^b^		Diploid^c^, 12 metacentric, 2 sub-metacentric chromosomes^d^; centromeric and telomeric sequence signals^e^	Zhang et al. (2012) ^a-e^
		C.sativusvar.sativus (CSS)		7^a^	14^b^		Diploid^c^, centromeric 45S (10) and intercalary 5S (2) rDNA signals^d^; centromeric and distal repetitive sequence probes^e^; molecular cytogenetic map^f^	Zhao et al. (2011)^b-e^, Yang et al. (2012)^b,f^, Zhang et al. (2016)^a-d^
		*C.sativus* cv. Winter Long			14^a^	7^b^	Diploid^c^, C- banding^d^, DAPI banding^e^, 45S (6) and 5 S (2) rDNA signals^f^, repetitive sequence based molecular karyotype in somatic and pachytene chromosomes^g^	Koo et al. (2002)^a-f^, (2005)^a,b,g^
		C.sativusvar.xishuangbannesis Qi et Yuan Zhenzhen, 1983		7^a^	14^b^		Diploid^c^, centromeric 45S (10) and intercalary 5S (2) rDNA signals^d^; centromeric and telomeric signals^e^	Zhao et al. (2011)^b,c,e^, Zhang et al. (2016)^a-d^
*C.setosus* Cogniaux, 1881					24^a^	12^b^	Diploid^c^; 4m+5sm+3st (haploid)^d^	Rajkumari et al. (2013)^a-c^, (2015)^a,c,d^
*C.silentvalleyii* Manilal et Sabu et Mathew, 1985					24^a^	12^b^	-	Rajkumari et al. (2013) ^a,b^
*C.trigonus* Roxb.					24^a^	12^b^	-	Rajkumari et al. (2013) ^a,b^
*C.zambianus* Widrl., J.H.Kirkbr., Ghebret. and K.R.Reitsma				12^a^	24^b^		Diploid^c^; 45S (2) and co-localized 45S+5S (2) signals^d^	Zhang et al. (2016) ^a-d^
*C.zeyheri* Sond.					24^a^, 48^b^		Diploid^c^, Allotetraploid^d^; 2C (Feulgen densitometry): 1.682^e^/2.846 pg^f^; 4 satellites^g^; 45S (2) and co-localized 45S+5S (2) rDNA signals^h^; FISH with bulked oligo probe from cucumber schromosome C7^i^; 24II^j^ , 12II^k^, 11II+2I^l^	IPCN^a,b,d,j,k,l^, Ramachandran and Narayan (1985)^a-f^, Han et al. (2015)^i^, Yagi et al. (2015)^a,c,g,h^
*Cucumellacinerea* (Cogn.) C.Jeffrey							2C (Feulgen Microdensitometry): 0.50pg^a^	Bennet et al. (1982)^a^
*Mukiamaderaspatana* (L.) M.Roem. (syn. *Cucumismaderaspatanas* and *Melothriamaderaspatana*)				12^a^	24^b^	11^c^, 12^d^	-	CCDB^b,c^, Rajkumari et al. (2015)^b,d^
*Oreosyceafricana* Hook.f. (syn. *Cucumissubsericeus*)				12^a^	48^b^		Tetraploid^c^; co-localized 45S and 5S rDNA signals (2)^d^; FISH with bulked oligo probe from cucumber chromosome C7^e^	Han et al. (2015)^e^, Zhang et al. (2016)^a-d^

#x: base number; 2n: zygotic number; n: gametic number; NOR: nucleolar organizing region; SAT: satellite chromosome; ScgCP: Single-copy gene-based chromosome painting (Lou et al. 2014); I: univalent, II: bivalent, IV: tetravalent; CCDB: Chromosome Counts Database; superscripts correspond to reference.

**Table 8.** Cytogenetic information in Cucurbiteae #.

Genera studied	Species studied	Chromosome no.			Ploidy, Genome size, Chromosome features	References
		x	2n	n		
*Cayaponia* Silva Manso, 1836	*C.laciniosa* Linnaeus, 1753		24^a^		-	Kumar and Subramaniam (1987) ^a^
*Cucurbita*		10^a^, 12^b^			-	Darlington and Janaki Ammal (1945) ^a,b^
	*C.andreana* Naudin, 1896		40^a^			CCDB ^a^
	*C.argyrosperma* Huber, 1867 (syn. *C.mixta* Pangalo, 1930)		40^a^		2C (flow cytometry): 0.748 pg^b^	Sisko et al. (2003)^a,b^
	*C.cylindrata* Bailey, 1943		40^a^	20^b^	-	CCDB ^a,b^
	*C.digitata* Gray, 1853	10^a^, 12^b^	40^c^	20^d^	-	Darlington and Janaki Ammal (1945)^a,b^, CCDB^c,d^
	*C.ecuadorensis* Cutler et Whitaker, 1969				2C: 0.72pg^a^	Plant DNA C Value database^a^
	*C.ficifolia* Bouché, 1837 (syn. *C.melanosperma* Gasparrini, 1847)		40^a^		2C (flow cytometry): 0.933pg^b^	Plant DNA C- Values Database^a,b^
	*C.foetidissima* Kunth, 1817	10^a^, 12^b^	40^c^, 42^d^		2C (flow cytometry): 0.686pg^e^	Darlington and Janaki Ammal (1945)^a,b^, Plant DNA C- Values Database^c,e^, CCDB^c,d^
	*C.indica* (unresolved)		40^a^		-	IPCN ^a^
	*C.lundelliana* Bailey, 1943			20^a^	2C (flow cytometry): 0.72pg^b^	CCDB^a^, Plant DNA C Value database^b^
	*C.maxima* Duchesne, 1786	20^a^	24^b^, 40^c^, 44^d^, 48^e^	20^f^		Kumar and Subramaniam (1987)^a,c,d,e^, Beevy and Kuriachan (1996)^f^, CCDB^c,f^
	*C.moschata* Duchesne, 1786	10^a^, 12^b^	24^c^, 40^d^, 44^e^,48^f^		Diploid^g^; 2C (Feulgen microdensitometry): 0.90pg^h^; 2C (flow cytometry): 0.708^i^/ 0.97^j^pg; 36 metacentric and 4 sub-metacentric chromosomes^k^; CSR: 1.05-1.78μm^l^, 45S (10) and 5S (4) rDNA signals^m^	CCDB^f^, Plant DNA C- Values Database^h,i^, Kumar and Subramaniam (1987)^a-f^, Barrow and Meister (2003)^j^, Xu et al. (2007)^d,m^, Waminal et al. (2011)^g,d,k,l,m^
	C.okeechobeensisssp.martinezii Bailey, 1943		40^a^		2C (flow cytometry): 0.74pg^b^	Plant DNA C- Values Database^a,b^
	*C.palmata* Watson, 1876	10^a^, 12^b^	40^c^, 42^d^	20^e^	-	Kumar and Subramaniam (1987)^a,b^, CCDB^c,d,e^
	*C.pedatifolia* Bailey, 1943		40^a^		-	CCDB ^a^
	*C.pepo* Linnaeus, 1753	10^a^, 12^b^	22^c^, 24^d^, 28^e^, 40^f^, 42^g^, 44^h^, 46^i^, 80^j^	20^k^	2C (flow cytometry): 0.74pg^l^; 0.864^m^; 1.109 pg-1.064 pg^n^; 1.18pg^o^; 45S (10) and 5S (4) rDNA signals^p^	Kumar and Subramaniam (1987)^a-j^, CCDB^f,k^, Marie and Brown (1993)^l^, Barow and Meister (2003)^o^, Rayburn (2008)^n^, Plant DNA C- Values Database^m^, Xie et al. (2019b)^f, p^
*Sicana* Naudin, 1862	*S.odorifera* Vellozo, 1831		40^a^	20^b^	-	IPCN ^a,b^

# x: base number; 2n: zygotic number; n: gametic number; CCDB: Chromosome Counts Database; superscripts correspond to references.

### ﻿Nuclear genome contents

Nuclear genome sizes are reported in 49 species (~5% of total species) belonging to 15 genera (~16% of total genera) of Cucurbitaceae. Among the understudied tribes, 2C genome content is known for one species each from Gomphogyneae and Coniandreae (Table 2). Within the Momordiceae species of India, significant interspecific genome size differences have been reported (Ghosh et al. 2021). The species differed 5.19-fold in their genome sizes (2C = 0.72-3.74 pg) (Table 3) (Ghosh et al. 2021). Interestingly, the species with lowest chromosome number (*M.cymbalaria*, 2n = 18) contained highest nuclear DNA content among the four *Momordica* species (Table 3). In Bryonieae, flow cytometric genome size of *Bryonia* shows a 2.2-fold increase than *Ecballium* (Table 4). In case of Sicyoeae, flow cytometric 2C DNA content ranges from 1.49–2.32 pg/2C, indicating 1.55-fold differences in genome size. *Echinocystislobata* Michaux, 1803, in spite of tetraploid condition, shows lowest genome size (Table 5). There is no significant difference in genome size between the genders of *Trichosanthesdioica* Roxburgh, 1832 (Table 5). Genome size estimates are known from 24 Benincaseae species of which 17 species belong to *Cucumis* (Tables 6, 7). Highest 2C nuclear genome is known in *Benincasahispida* Thunberg, 1784 (1.97 pg) (Bhowmick and Jha 2015a) while the lowest is known in Cucumismelovar.inodorus Harz, 1885 (0.64 pg) (Karimzadeh et al. 2010). In case of *Cucumis*, there is yet no consensus on whether the taxa with different base numbers (x = 7, 12) have correspondingly dissimilar genome sizes since the researchers depended on diverse methods of genome size estimation. Lower 2C genome size was reported in *C. Cocciniagrandis* Linnaeus, 1767 (2n = 24) while *C.trilobata* (2n = 20) had higher 2C DNA content (Table 6). The divergence in genome size between genders was found to be highest in dioecious *C.grandis* (Table 6), a sharp contrast to dioecious *Trichosanthesdioica* (Table 5). Benincaseae shows a 3.07-fold overall difference in genome size. Genome sizes are known in eight species of *Cucurbita* Jussieu, 1789. Flow cytometric genome size ranges from 0.686–0.933 pg/2C, indicating a 1.36-fold variation (Table 8). Despite polypoidy, the nuclear DNA content of *Cucurbita* species is comparable to many diploids.

### ﻿Karyotypes, chromosome banding and molecular cytogenetics

Among the understudied tribes, information on chromosome morphology, size and karyotype are reported in very few taxa (Table 2). In *Gynostemmapentaphyllum* Thunberg, 1784, the number of rDNA loci was suggested to reduce during polyploidization (Pellerin et al. 2018). The *Actinostemmatenerum* Griffith, 1837, genome contained interstitial telomeric repeats which were suggested to be the result of chromosome fusion from ancestral genome. The co-localization of 45S and 5S rDNA loci in *A.tenerum* and *Thladianthadubia* Bunge, 1833, have been thought to imply regional synteny and shared ancestral traits (Xie et al. 2019b). In the tribe Cucurbiteae, detailed karyotype analysis is known only in *Cucurbitamoschata* Duchesne, 1786 and *C.pepo* Linnaeus, 1753, showing conserved 45S and 5S rDNA signals (non-co-localized) in independent analyses (Table 8).

Karyotypes and chromosome sizes are reported in ten species of Momordiceae (Table 3). Interspecific differences have been observed and found to correlate with phylogenetic relationship within *Momordica* (Ghosh et al. 2021). Infraspecific delimitation of Indian *M.charantia* varieties was based on fluorochrome banding pattern and genome size divergence (Table 3), corresponding to infraspecific distinction reported in the Japanese bitter gourd cultivars (Kido et al. 2016). FISH in three *Momordica* species revealed 45S and 5S rDNA sites to be localised on different chromosomes (Table 3). In context of the genome sequence of bitter gourds (Matsumura et al. 2020), further scopes for cytogenetic and genomic investigation remain open.

Karyotype and chromosome size is reported in eight 8 species of Sicyoeae (Table 5). Fluorochrome banding pattern has facilitated comparative analysis in *Luffa* species occurring in India (Tables 1, 5) (Bhowmick and Jha 2015a, 2021). The cultivated ridged gourd (*L.acutangula* Linnaeus, 1753) showed three CMA^+^ satellite bearing pairs (Fig. 1A–C, J) as in the wild *L.echinata* Roxburgh, 1814 (Fig. 1G–I, L), while the sponge gourd (*L.aegyptiaca* Miller, 1768 has two satellited pairs (Fig. 1D–F, K). *Luffaacutangula* and *L.echinata* also showed up distal DAPI bands (Fig. 1J, L), absent in *L.aegyptiaca* (Fig. 1K). *Trichosanthes* species (2n = 22) have inter-specific differences (Fig. 2) as well as infraspecific distinction (*T.cucumerina* Linnaeus, 1753) in fluorochrome banding pattern (Tables 1, 5, Fig. 2A–H). The male and female plants of *T.dioica* show similar chromosome number, morphology and genome size but show differences in fluorochrome banding pattern (Fig. 2I–P, Table 5). The 11^th^, 12^th^ and 13^th^ pairs (CMA^+^) are marker chromosomes in *Luffa* (Fig. 1, Table 1) while the 10^th^ and 11^th^ pairs are conserved CMA^+^ satellited pairs in *Trichosanthes* (Fig. 2, Table 1). Eight species of Sicyoeae have been subjected to FISH (Table 5). The polyploid and diploid species have differences in the number of rDNA loci, showing separate localization of the 45S and 5S rDNA signals except *Sicyosangulatus* Linnaeus, 1753 and *Trichosantheskirilowii* Maximowicz, 1859 (Table 5).

Benincaseae generally reveal two distal 45S rDNA loci of which at least one locus is either adjacent to 5S rDNA locus (Table 6) or co-localized in the same chromosome as in most of the *Cucumis* species (Table 7). Exceptionally, a wild species of *Benincasa* (*B.fistulosa* Stocks, 1851) has non-adjacent 45S and 5S signals (Li et al. 2016). GC rich satellites were observed in the 12^th^ pair of chromosomes showing CMA^+^ bands in cultivated Indian ashgourd (*B.hispida*) (Fig. 3 A–C, J, Tables 1, 6). *Lagenariasiceraria* Molina, 1782 and *Cucumismelo* Linnaeus, 1753 are the other two genera having similarity in rDNA hybridization profile, agreeing with phylogenetic affinity (Li et al. 2016).

*Citrulluscolocynthis* Linnaeus, 1753 and *C.lanatus* Thunberg, 1794 may share a common ancestor both having two 45S rDNA loci and one 5S locus. Loss of one 45S rDNA locus has given way to *C.rehmii* De Winter, 1990 while gain of one 5S rDNA locus has been proposed to lead to *C.ecirrhosus* Cogniaux, 1888 and C.lanatusvar.citroides Bailey, 1930 (presently *C.amarus* Schrader, 1836) (Reddy et al. 2013; Li et al. 2016). GISH using C.lanatusvar.citroides genome has revealed divergence from C.lanatusvar.lanatus (Reddy et al. 2013).

The genus *Cucumis* is the largest in Benincaseae with 65 species of which 39 have been studied (Table 7). Among the *Cucumis* species with x = 12, co-localization rDNA loci (45S and 5S rDNA) have been documented in 14 species, including *C.melo* (Table 7). However, the number of 45S sites is generally four, which may be six or eight in some cases (Table 7). rDNA hybridization data strongly corroborated with the ‘fusion’ theory for derivation of x = 7 (*C.sativus*) from x = 12 (*C.melo*) (Waminal and Kim 2012) which is substantiated by genomic studies (Li et al. 2011). There are ten pericentromeric/ centromeric 45S and two distal 5S rDNA sites in *C.sativus* while six 45S rDNA sites were reported in C.sativusvar.hardwickii Royle, 1835 (Koo et al. 2005; Zhang et al. 2012). Comparative chromosome painting (Lou et al. 2014) and GISH (Zhang et al. 2015) proved high colinearity between cucumber and melon. Based on chloroplast and nuclear DNA (ITS) phylogeny, *C.melo* (melon) has been found to be sister to a clade comprising *C.sativus* and related genera (*Dicaelospermum* Clarke, 1879 and *Mukia* Arnott, 1840) (Renner et al. 2007). rDNA site co-localization was found to coincide with geographical origin of 12 *Cucumis* species (Zhang et al. 2016). The chromosomal affinity between *C.metuliferus* Schrader, 1838, *C.anguira* Linnaeus, 1753, *C.zeyheri* Sonder, 1862, *C.myriocarpus* Naudin, 1859 and polyploid *C.heptadactylis* Naudin, 1859 (dioecious) (Yagi et al. 2015) can be substantiated by their phylogenetic proximity based on chloroplast and nuclear DNA (ITS) sequences (Renner et al. 2007). rDNA distribution of *C.metuliferus* was also the reason to consider proximity with *Citrullusnaudinianus* Sonder, 1862, (previously *Acanthosicyosnaudinianus* Sonder, 1862) (Reddy et al. 2013). Infraspecific differences were documented in *Cucumismelo* on the basis of 45S- 5S rDNA signals (linked or separated) which also possessed unique centromeric satellites (Setiawan et al. 2018, 2020). Moreover, chromosome painting method elucidated chromosomal rearrangement in some *Cucumis* species (Lou et al. 2014; Li et al. 2018).

The dramatic evolution of Y chromosome was validated in karyotypes (Fig. 3 D–I, K–L) of *Cocciniagrandis* (Table 6). The 45S rDNA sites enabled confirmation of NORs in the 8^th^ and 12^th^ pair containing distal GC rich CMA^+^ signals in *C.grandis* (Fig. 3 D–I, K–L, Tables 1, 6). 45S and 5S rDNA hybridization pattern was similar in three other *Coccini*a species and *Diplocyclospalmatus* Linnaeus, 1753 (Table 6). The three closely related dioecious species of *Coccinia* accumulated Y chromosome repeats and displayed sex chromosome turnover (Sousa et al. 2017). Strong centromeric CMA bands (Fig. 3 D–I, K–L, Table 1) were observed in *C.grandis* except Y chromosome (Fig. 3 I, L), presenting a possibility that *CgCent* (CL1) is a feature of centromeres of dioecious *Coccinia* species (Sousa et al. 2017). In addition, non-nucleolar CMA^+^ heterochromatin might be associated with sexual differentiation of autosomes in dioecious *C.grandis* (Fig. 3) which is also a marker in *Trichosanthesdioica* (Fig. 2, Table 1), opening good scope for further study.

**Figure 4.**
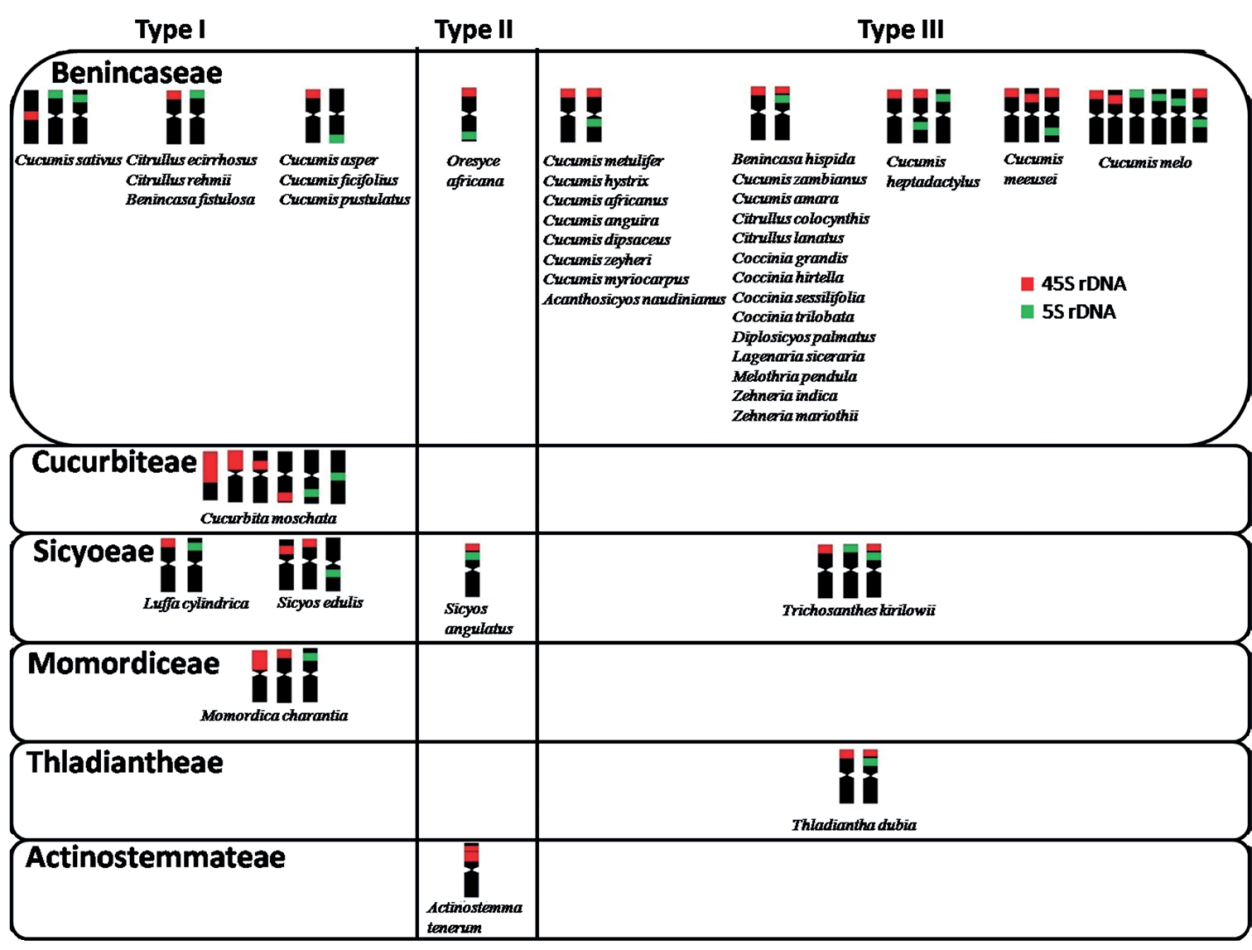
Types of chromosomes bearing the NORs as per available reports of rDNA hybridization in Cucurbitaceae. Type I: Chromosomes with only non-colocalised 45S and 5S rDNA sites, Type II: Chromosomes with colocalised 45S and 5S rDNA sites, Type III: Chromosomes with both non- colocalised and colocalised 45S and 5S rDNA sites. See text for explanation.

Distinct 45S rDNA sites are higher in number than 5S rDNA sites in Cucurbitaceae (Fig. 4) (Waminal and Kim 2012). The distal 45S rDNA loci are conserved genomic landmarks (Fig. 4) while 5S rDNA loci are relatively diverse (Fig. 4). Based on the literature reports, some NORs (Type I) included chromosomes showing non-colocalized 45S and 5S rDNA sites in seven species of Benincaseae, one species each from Cucurbiteae and Momordiceae and two species of Sicyoeae. The rearrangement of 45S rDNA site in *Cucumissativus*, probes for chromosome number reduction which may be a consequence of diploidization. The second type (Type II) shows colocalised 45S and 5S rDNA loci, either adjacent or distant, but always on the same chromosome and found in one species each of Benincaseae, Sicyoeae and Actinostemmateae. The third type (Type III) was characterized by chromosomes with non-colocalized and colocalised 45S and 5S rDNA loci, as in 14 species of Benincaseae and one species each of Sicyoeae and Thladiantheae. The rDNA sites of majority of *Cucumis* species were of non-adjacent type. Hence, type III NORs in majority of Benincaseae genera advocates conservation of the marker chromosomes having distal NOR (45S rDNA). *Gynostemmapentaphyllum* and some polyploid *Cucumis* reveal rDNA loci reduction after polyploidization (Zhang et al. 2016; Pellerin et al. 2018).

### ﻿Correlation between parameters

**Figure 5.**
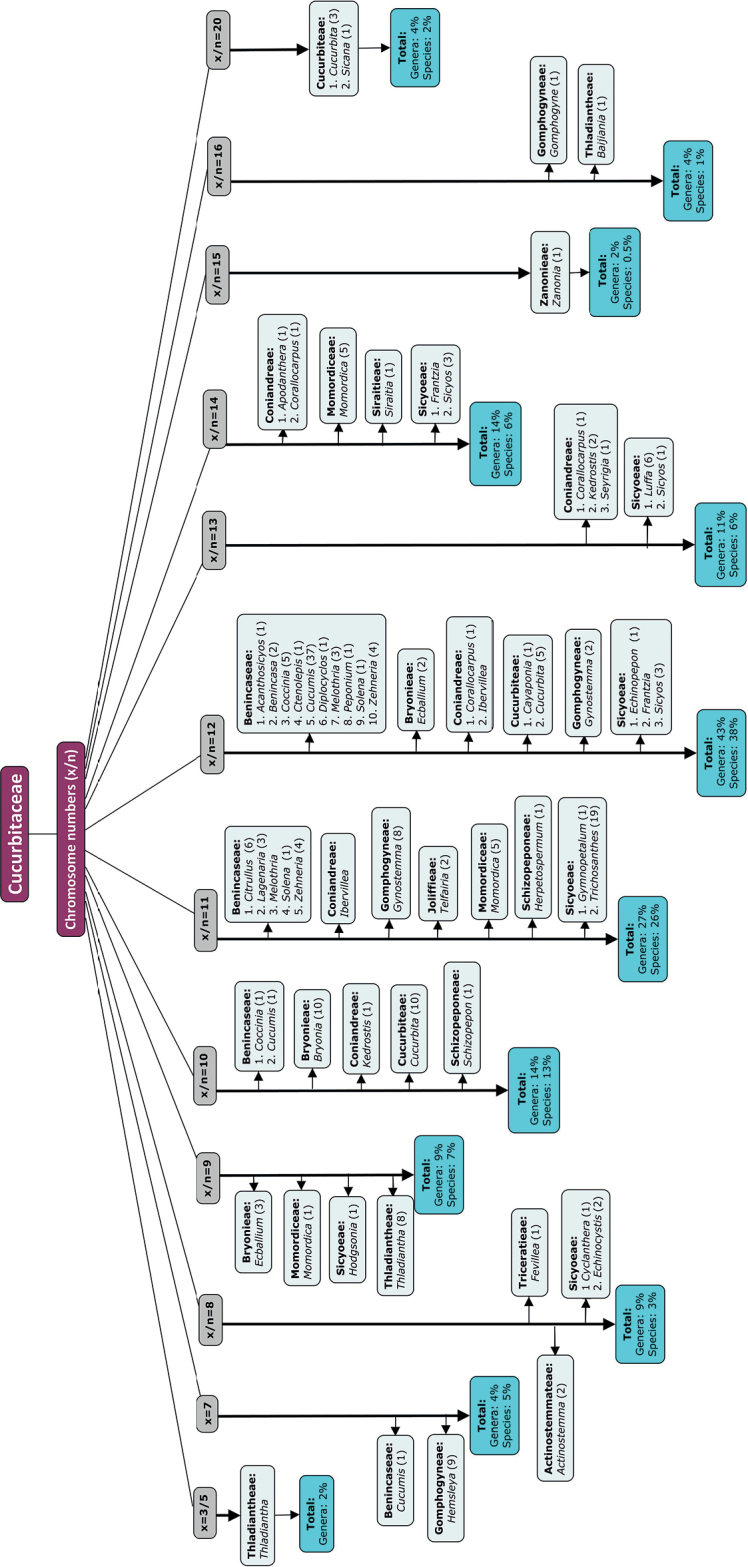
The types of different base numbers (x, based on published reports) or possible base numbers (x/n, based on reported haploid counts) in Cucurbitaceae. The numbers in brackets beside names of genera signify the number of species whose chromosome counts are reported. The % of genera and species with a particular chromosome number, is indicated at the end arrow (out of a total of 44 genera and 188 species with chromosome counts).

**Figure 6.**
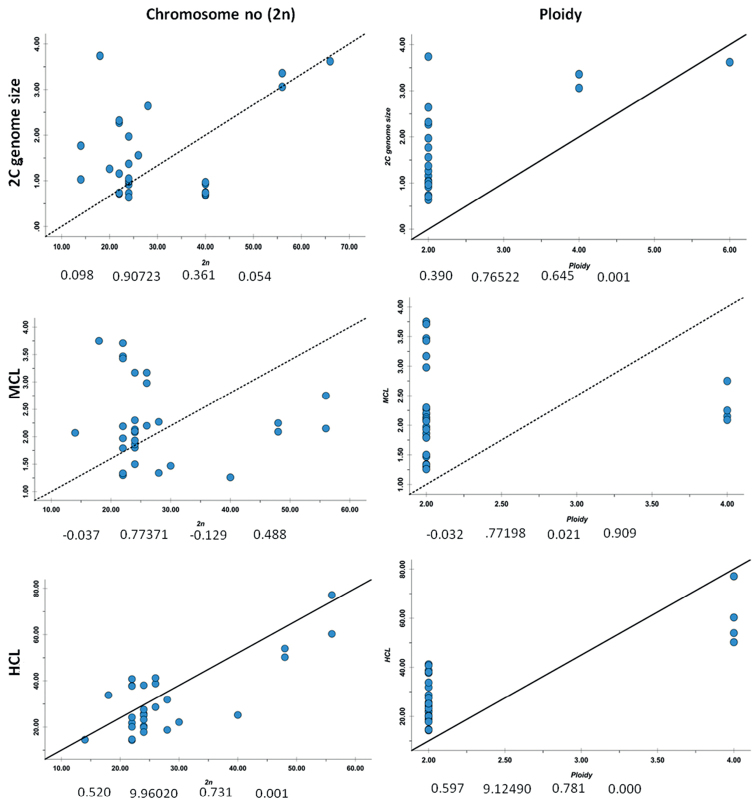
Scatter plots of 2n chromosome number and ploidy level (predictor variables) versus 2C genome size, MCL (mean chromosome length) and HCL (total length of haploid chromosome set) in Cucurbitaceae taxa. Symbols below plots depict regression analysis parameters; square: adjusted R square, circle: standard error of the estimate, triangle: Pearson Correlation, star: 2-tailed significance of Pearson Correlation. Regular lines indicate significant linear regression and dotted lines indicate not significant linear regress

Chromsome numbers in Cucurbitaceae range from x = 5 to x = 16. The most prevalent number x = 12 (Fig. 5) is considered ancestral (Xie et al. 2019b), followed by x = 11, 13, 14 and 10 (Fig. 5). The present regression analyses for 41 taxa (including 16 Indian taxa) (Table 9) revealed significant linear correlation between 2n and HCL, between ploidy and genome size and between ploidy and HCL (Fig. 6). Therefore, an increase in ploidy/ 2n number is linked with increase in HCL. There was no significant correlation between 2C genome size and chromosome numbers. Cytogenetic parameters may not reflect residual evidence of CCT in Cucurbitraceae at present, as reasoned by Alix et al. (2017).

**Table 9.** Data on fundamental cytogenetic parameters utilized for statistical analysis.

Species	2n Chromosome no.	Ploidy	2C genome size (pg)	MCL (μm)	HCL (μm)	References
*Gynostemmapentaphyllum*	66	6	3.62			Zhang et al. (2013), Pellerin et al. (2018)
*Zanoniaindica*	30	2		1.47	22.12	Lekhak et al. (2018)
*Momordicabalsamina*	22	2		1.30	14.3#	Bharathi et al. (2011)
Momordicacharantiavar.charantia	22	2	0.72	1.97	21.77	Ghosh et al. (2018)
Momordicacharantiavar.muricata	22	2	1.16	2.19	24.19	Ghosh et al. (2018)
*Momordicacochinchinensis*	28	2	2.64	2.27	31.86	Ghosh et al. (2021)
*Momordicacymbalaria*	18	2	3.74	3.75	33.79	Ghosh et al. (2021)
*Momordicadioica*	56	4	3.36	2.75	77.1	Ghosh et al. (2021)
*Momordicasahyadrica*	28	2		1.34	18.76	Bharathi et al. (2011)
*Momordicasubangulata*	56	4	3.06	2.15	60.3	Ghosh et al. (2021)
*Luffaacutangula*	26	2		2.20	28.63	this study
*Luffacylindrica*	26	2	1.56	2.98	38.77	Bhowmick and Jha (2015a), this study
*Luffaechinata*	26	2		3.17	41.26	this study
*Trichosanthescucumerina*	22	2		3.47	37.855	Bhowmick and Jha (2019), this study
Trichosanthescucumerinasubsp.cucumerina Anguina	22	2		3.43	37.74	Bhowmick and Jha (2019), this study
*Trichosanthesdioica Male*	22	2	2.27	3.71	40.82	Bhowmick and Jha (2015a), this study
*Trichosanthesdioica Female*	22	2	2.32	3.71	40.82	Bhowmick and Jha (2015a), this study
*Benincasahispida*	24	2	1.97	3.17	38.08	Bhowmick and Jha (2015a), this study
*Citrulluslanatus*	22	2		1.33#	14.67	Waminal et al. (2011)
*Cocciniagrandis* male	24	2	0.92	1.80	20.32	Bhowmick et al. (2012, 2016), this study
*Cocciniagrandis* female	24	2	0.73	1.86	19.85	Bhowmick et al. (2012, 2016), this study
*Cocciniahirtella*	24	2	0.988			Sousa et al. (2017)
*Cocciniasessilifolia* Male	24	2	0.984			Sousa et al. (2017)
*Cocciniasessilifolia* Female	24	2	0.998			Sousa et al. (2017)
*Cocciniatrilobata*	20	2	1.263			Sousa et al. (2017)
*Lagenariasiceraria*	22	2	0.734	1.79	20.06	Achigan-Dako et al. (2008)
*Cucumisafricanus*	24	2		2.08	25.045	Yagi et al. (2015)
Cucumisanguriavar.anguria	24	2		2.13	25.6	Yagi et al. (2015)
Cucumisanguriavar.longaculeatus	24	2		2.10	25.195	Yagi et al. (2015)
*Cucumisheptadactylus*	48	4		2.09	50.225	Yagi et al. (2015)
*Cucumismelo*	24	2	1.05	1.50	17.8#	Marie and Brown (1993), Hoshi et al. (2013)
Cucumismelovar.inodorus	24	2	0.64			Karimzadeh et al. (2010)
Cucumismyriocarpusvar.leptodermis	24	2		1.93	23.19	Yagi et al. (2015)
Cucumismyriocarpusvar.myriocarpus	48	4		2.25	53.985	Yagi et al. (2015)
*Cucumiszeyheri*	24	2		2.30	27.56	Yagi et al. (2015)
*Cucumissativus*	14	2	1.03, 1.77##	2.07#	14.50	Barow and Meister (2003), Marie and Brown (1993), Waminal and Kim (2012)
*Cucurbitaargyrosperma*	40		0.748			Roy et al. (1991), Sisko et al. (2003)
*Cucurbitaecuadorensis*	40		0.933			Sisko et al. (2003)
*Cucurbitafoetidissima*	40		0.686			Sisko et al. (2003)
*Cucurbitamoschata*	40	2	0.708, 0.97##	1.26#	25.19	Sisko et al. (2003), Barrow and Meister (2003), Waminal et al. (2011)
Cucurbitaokeechobeensisssp.martinezii	40		0.74			Sisko et al. (2003)

# calculated from chromosome measurements reported in publications, ## different entries for same taxa were taken from different reports

## ﻿Future directions

Chromosome number and genome size information in the basal clades (understudied tribes) should be given attention to infer ancient base numbers. The parameters of fundamental and molecular cytogenetics are inevitable for genomic interpretation (Weiss-Schneeweiss and Schneeweiss 2013; Deakin et al. 2019) and hence relevant to spot genetic resources and relationships with wild relatives. The current review is not exhaustive but supersedes the scopes of general web resources and brings an offline resource exclusive for Cucurbitaceae.
