# ﻿Corrigenda: ﻿Bhowmick BK, Jha S (2022) ﻿A critical review on cytogenetics of Cucurbitaceae with updates on Indian taxa. Comparative Cytogenetics 16(2): 93–126. https://doi.org/10.3897/compcytogen.v16.i2.79033

**DOI:** 10.3897/compcytogen.19.164044

**Published:** 2025-07-25

**Authors:** Biplab Kumar Bhowmick, Sumita Jha

**Affiliations:** 1 Department of Botany, Scottish Church College, 1&3, Urquhart Square, Kolkata-700006, West Bengal, India Scottish Church College Kolkata India; 2 Plant Cytogenetics and Biotechnology Laboratory, Department of Botany, University of Calcutta, 35, Ballygunge Circular Road, Kolkata 700019, West Bengal, India University of Calcutta Kolkata India

After the publication of our article, we detected an error in Fig. 2. Image 2M represents DAPI-stained somatic chromosomes of *Trichosanthesdioica* (female), which has been duplicated in panel 2C due to an inadvertent mistake during figure assembly. Hence, the correct Fig. 2C for DAPI-stained somatic chromosomes of Trichosanthescucumerinassp.cucumerina has been inserted in the corrected Fig. 2.

The corrected Fig. 2 and its accompanying legend appear below.

Two additional typographical errors were noted, one in Table 1 and the other in the legend of Fig. 1.

In Table 1, *Luffacylindricaaegyptiaca* Miller, 1768 should be *Luffaaegyptiaca* Miller, 1768.

In Figure 1 legend D-F, *L.aegyptiacacylindrica* should be *L.aegyptiaca*.

**Figure 2.**
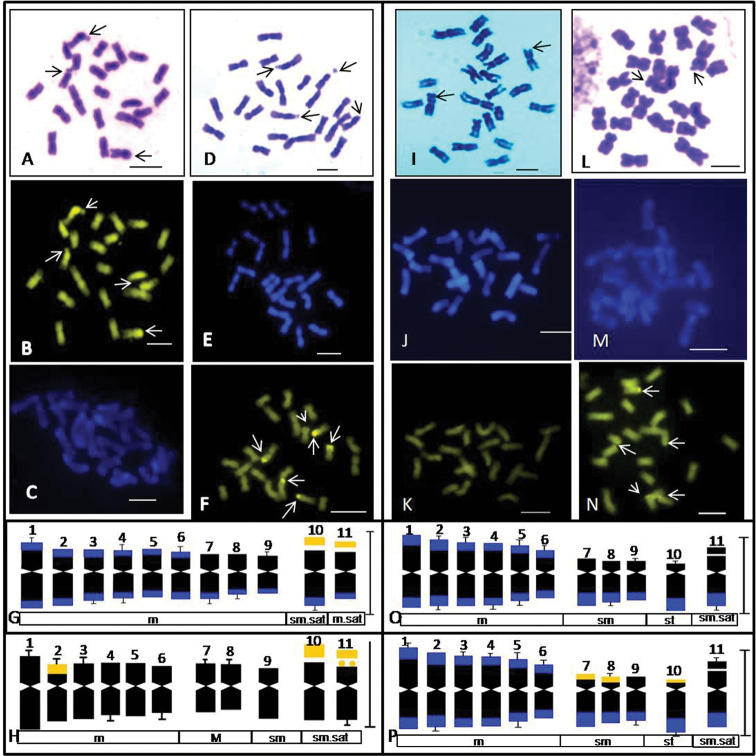
Somatic metaphase chromosomes and idiograms of *Trichosanthes* species stained with Giemsa (**A, D, I, L**), DAPI (**C, E, J, M**) and CMA3 (**B, F, K, N**) **A–C.**T.cucumerinassp.cucumerina (2n = 22), **D–F.**Trichosanthescucumerinassp.cucumerina ‘Anguina’ (2n = 22) **I–K.***T.dioica* (male, 2n = 22) **L–N.***T.dioica* (female, 2n = 22). Arrows indicate satellited chromosomes in Giemsa plates and CMA^+ve^ signals in **B, F, K, N**. Corresponding somatic idiograms (haploid set) of: **G**T.cucumerinassp.cucumerina**H.**Trichosanthescucumerinassp.cucumerina ‘Anguina’ **O.***T.dioica* male plant **P.***T.dioica* female plant. Blue and golden yellow bands in idiograms indicate DAPI^+ve^ and CMA^+ve^ signals, respectively. Scale bars: 5 μm.

